# Derivation and characterisation of endothelial cells from patients with chronic thromboembolic pulmonary hypertension

**DOI:** 10.1038/s41598-021-98320-1

**Published:** 2021-09-22

**Authors:** Olga Tura-Ceide, Valérie F. E. D. Smolders, Núria Aventin, Constanza Morén, Mariona Guitart-Mampel, Isabel Blanco, Lucilla Piccari, Jeisson Osorio, Cristina Rodríguez, Montserrat Rigol, Núria Solanes, Andrea Malandrino, Kondababu Kurakula, Marie Jose Goumans, Paul H. A. Quax, Victor I. Peinado, Manuel Castellà, Joan Albert Barberà

**Affiliations:** 1 Department of Pulmonary Medicine, Servei de Pneumologia, Hospital Clínic-Institut d’Investigacions Biomèdiques August Pi I Sunyer (IDIBAPS), University of Barcelona, Villarroel, 170, 08036 Barcelona, Spain; 2 Biomedical Research Networking Centre on Respiratory Diseases (CIBERES), 28029 Madrid, Spain; 3 Department of Pulmonary Medicine, Santa Caterina Hospital de Salt and the Girona Biomedical Research Institut (IDIBGI), Dr. Josep Trueta University Hospital de Girona, 17190 Girona, Spain; 4 Department of Biochemistry and Molecular Biology, Faculty of Biology, University of Barcelona, Barcelona, Spain; 5 Department of Vascular Surgery, Leiden University Medical Center, Einthoven Laboratory for Experimental Vascular Medicine, Leiden University Medical Center, Leiden, The Netherlands; 6 Laboratory of Muscle Research and Mitochondrial Function, Department of Internal Medicine, Hospital Clínic of Barcelona (HCB), Institut d’Investigacions Biomèdiques August Pi I Sunyer (IDIBAPS), University of Barcelona (UB), Barcelona, Spain; 7 Biomedical Research Networking Centre on Rare Diseases (CIBERER), Madrid, Spain; 8 Cardiovascular Institute, Hospital Clínic de Barcelona-Institut d’Investigacions Biomèdiques August Pi I Sunyer (IDIBAPS), University of Barcelona, Barcelona, Spain; 9 Biomedical Research Networking Center on Cardiovascular Diseases (CIBERCV), Madrid, Spain; 10 European Molecular Biology Laboratory (EMBL), Institute for Bioengineering of Catalonia (IBEC), Barcelona, Spain; 11 Department of Cell and Chemical Biology, Leiden University Medical Center, Leiden, The Netherlands; 12 Department of Cardiovascular Surgery, Cardiovascular Institute, Hospital Clínic, University of Barcelona, Barcelona, Spain

**Keywords:** Mechanisms of disease, Translational research, Cardiovascular diseases

## Abstract

Pulmonary endarterectomy (PEA) resected material offers a unique opportunity to develop an in vitro endothelial cell model of chronic thromboembolic pulmonary hypertension (CTEPH). We aimed to comprehensively analyze the endothelial function, molecular signature, and mitochondrial profile of CTEPH-derived endothelial cells to better understand the pathophysiological mechanisms of endothelial dysfunction behind CTEPH, and to identify potential novel targets for the prevention and treatment of the disease. Isolated cells from specimens obtained at PEA (CTEPH-EC), were characterized based on morphology, phenotype, and functional analyses (in vitro and in vivo tubule formation, proliferation, apoptosis, and migration). Mitochondrial content, morphology, and dynamics, as well as high-resolution respirometry and oxidative stress, were also studied. CTEPH-EC displayed a hyperproliferative phenotype with an increase expression of adhesion molecules and a decreased apoptosis, eNOS activity, migration capacity and reduced angiogenic capacity in vitro and in vivo compared to healthy endothelial cells. CTEPH-EC presented altered mitochondrial dynamics, increased mitochondrial respiration and an unbalanced production of reactive oxygen species and antioxidants. Our study is the foremost comprehensive investigation of CTEPH-EC. Modulation of redox, mitochondrial homeostasis and adhesion molecule overexpression arise as novel targets and biomarkers in CTEPH.

## Introduction

Chronic thromboembolic pulmonary hypertension (CTEPH) is a severe cause of pulmonary hypertension (PH)^1^, defined by increased mean pulmonary artery pressure due to non-resolved thrombotic lesions in pulmonary arteries despite appropriate anticoagulant therapy^2^. It is a progressive disease with significant burden in terms of severity and prevalence^1,3^. CTEPH may develop in 3–4% of cases after an acute pulmonary embolism and is mostly underdiagnosed^4^. Chronic obliteration of pulmonary arteries by the presence of an intraluminal organized thrombi, produces a gradual increase in pulmonary vascular resistance (PVR), leading to right ventricular failure and death^5^. The pathogenesis of CTEPH, the mechanisms leading to the lack of thrombus resolution and the development of peripheral vasculopathy are still unknown. A better understanding of how pulmonary endothelial dysfunction contributes to the pathogenesis of CTEPH might improve the therapeutic management of the disease.

Pulmonary endarterectomy (PEA), which consists in the surgical removal of the occluding thromboembolic material from pulmonary arteries, is the treatment of choice for CTEPH^6^. PEA provides symptomatic, hemodynamic, and prognostic benefit^1,7^. However, up to 50% of patients are not eligible for surgery and up to 35% of operated patients show persistent or residual PH^8^.

Endothelial dysfunction is believed to be an initial and essential step in the onset and progression of pulmonary arterial hypertension (PAH)^9^. Previous studies in CTEPH have also shown an hyperproliferative dysfunctional phenotype in CTEPH endothelial cells pointing out to its potential contribution in the progression of unresolved thrombi and vascular remodeling^10,11^. However, no well characterized biomarkers for endothelial dysfunction have been identified and/or translated into the clinical practice as useful tools to detect its presence and severity in CTEPH. In 1973 *Moser and Braunwald*, examining the histopathological features of PEA specimens, found distinct vessel abnormalities such as media thickening and increased intimal cell proliferation^12^. Interestingly, Quarck et al., analyzing the composition of PEA tissues, observed thrombus recanalization and vascular neoangiogenesis that correlated with patient’s outcome^13^. Deficient angiogenesis, particularly lower proportion of mature neovessels, were suggested to be key in the progression of the disease. Additionally, it has been recently shown that CTEPH pulmonary arteries presented an abnormal vasodilator response to acetylcholine indicating the existence of an endothelial dysfunction in CTEPH^14^.

Intravascular occluding material extracted during PEA offers a unique opportunity to evaluate patient-specific ECs at the disease site. In this study, we hypothesized that CTEPH-pulmonary endothelial cells (ECs) present phenotypic, angiogenic and mitochondrial abnormalities that are critical for the development and progression of CTEPH. We also hypothesize that homeobox (HOX) genic expression pattern is a good marker to understand the origin of the CTEPH isolated ECs.

Accordingly, we aimed to develop an in vitro EC model of CTEPH to determine its functional characteristics, molecular signature, and mitochondrial profile, and to identify potential key targets and molecular pathways for CTEPH prevention and treatment.

## Materials and methods

An expanded material and methods section is available in the supplementary document.

### Subjects

Subjects with CTEPH diagnosed per current guidelines^15^, who underwent PEA at the Hospital Clinic of Barcelona, Spain were enrolled. Patient characteristics are shown in supplementary Table 1.

### Morphometric and histological assessments

PEA resected material (supplementary Fig. 1) was fixed and stained. Cellular markers were analyzed by immunohistochemistry as described^16^. On the other hand, PEA material was digested, stained, and analyzed by flow cytometry. The antibodies used are listed in supplementary Table 2.

### Primary cell cultures

Isolated ECs (CTEPH-EC) were obtained from PEA specimens. Human pulmonary artery smooth muscle cells (SMCs), human lung microvascular ECs (HMVEC-L) and human pulmonary artery ECs (HPAE) were used as control cells (Lonza).

### Cell characterization

Cells were directly analyzed by flow cytometry and immunofluorescence for phenotypic expression and proliferation. Total RNA and protein extraction were performed following manufacturer’s instructions. Primer sequences and antibodies are listed in supplementary Tables 2 and 3.

### Cell growth kinetics and cell viability

Fold expansion/day was measured as number of final cells divided by the number of seeded cells/days of culture. Cellular viability was determined using Vybrant MTT Cell Proliferation Kit (ThermoFisher Scientific). For clonogenic assays, single cells were plated in a 96-well plate and cultured as previously described^17^. Cells were also loaded into the xCELLigence device following manufacturer’s instructions to measure cell proliferation. Cellular circumference, area and diameter were measured using freely available imaging processing ImageJ software, version:2.1.0/1.53c, http://imagej.net/contributors.

### Tube formation assay and wound healing

ECs were seeded in an ibiTreat μ-Slide Angiogenesis (Ibidi) following manufacturer’s instructions. 3D microvascular networks (μVN) were obtained by a microfluidic approach. Wound closure was expressed as % of wound healed divided by area and width of original wound.

### Subcutaneous sponge implantation assay for in vivo vascularization

Non-obese diabetic (NOD-SCID)-IL-2 gammaRnull mice were used. Each animal had a control vehicle-impregnated sponge implanted on one flank and cell-impregnated sponge on the other flank. Sponges were excised 21 days following implantation, fixed, and stained for identification of blood vessels. All procedures were conducted following the European Directive 2010/63/UE and Spanish RD 53/2013 regulations related to the Guide for the Care and Use of Laboratory Animals and in compliance with the ARRIVE guidelines. The study protocol was approved by the Animal Experimentation Ethics Committee of the University of Barcelona (DAAM 10,028).

### Electron microscopy

CTEPH-EC or HPAE were fixed with 2.5% (w/v) glutaraldehyde in 0.1 M cacodylate buffer. Cell pellets were stored and analyzed by the technologic center of University of Barcelona.

### High resolution respirometry

CTEPH-EC or HPAE were resuspended in MiR05 medium and introduced into Oxygraph-2 k (Oroboros Instruments). Endogenous cell respiration and complex I-IV analysis using specific substrates and inhibitors were performed (detailed in supplementary Table 4).

### Mitochondrial morphology and content

Mitochondrial morphology and content were determined using confocal microscopy and MitoTracker green following manufacturer´s instructions.

### Detection of oxidative stress

Cellular and mitochondrial oxidation was measured using cell-permeant CellROX and MitoSOX (ThermoFisher Scientific). Total oxidized proteins were measured with the Oxyblot Protein Oxidation Kit (Merck Millipore) following manufacturer’s instructions.

### Statistical analysis

Statistical analyses were performed using GraphPad Prism 7 software, version 7.0e, serial number:GP7-0633739-R###-#####, https://www.graphpad.com. Data are shown as mean ± standard deviation. Independent samples were analyzed using the unpaired Student’s t-test (Mann–Whitney U test) to compare differences between two independent groups. More than two groups were compared using One-way ANOVA with Tukey´s post-hoc test or non-parametric analysis of variance Kruskal–Wallis test with a Dunn´s post-hoc multiple comparison test. The Spearman rank correlation coefficient was used as a hypothesis test to study the dependence between two random variables. Statistical significance was assumed if a null hypothesis could be rejected at *p* ≤ 0.05 (for a confidence interval of a = 95%).

### Ethical statement

The study was conducted in accordance with the Declaration of Helsinki, was approved by the Committee on Human Research of the Hospital Clínic of Barcelona and all subjects gave written informed consent. The study protocol was approved by the Animal Experimentation Ethics Committee of the University of Barcelona (DAAM 10028).

### Ethics approval

All procedures performed in studies involving human participants were in accordance with the ethical standards of the institutional and/or national research committee and with the 1964 Helsinki Declaration and its later amendments or comparable ethical standards. The study protocol was granted by the Hospital Clínic of Barcelona ethics committee (HCB/2018/0837 and HCB/2018/0434). The study protocol was approved by the Animal Experimentation Ethics Committee of the University of Barcelona (DAAM 10028).

### Consent to participate

All samples were collected with written informed consent of the participants.

## Results

### Histological assessment of resected specimens at pulmonary endarterectomy

**Figure 1:**
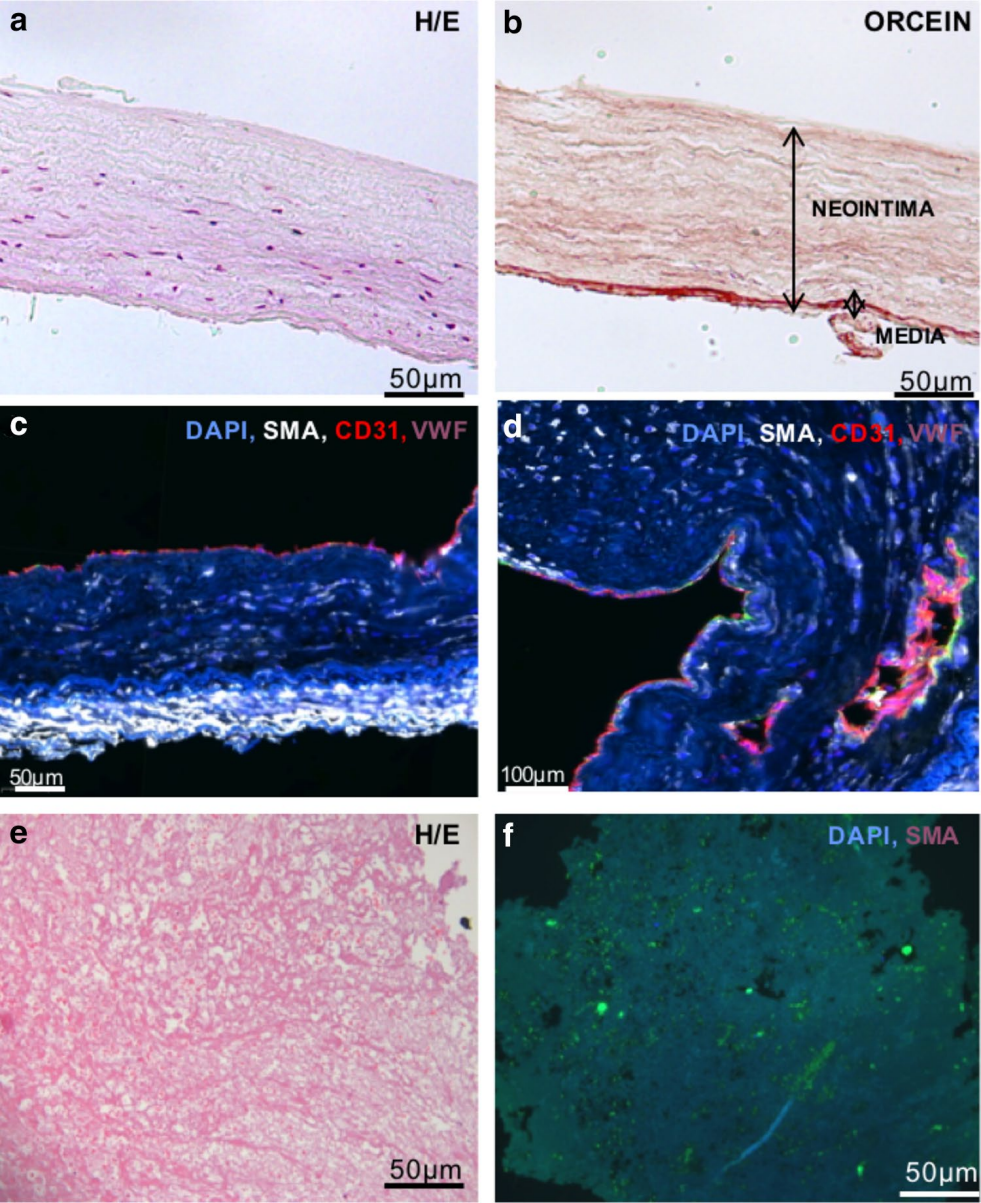
PEA samples. (**a**, **b**) Specimens collected during PEA from CTEPH patients were stained by hematoxylin and eosin and stained for elastin by orcein stain. (**c**) PEA samples presented an intact endothelium as shown by endothelial markers CD31 and vWF, an enlarged α-SMA negative neointima and an α-SMA positive organized media. (**d**) PEA samples showed the presence of mature microvessels. (**E**–**f**) Coagulant material showed a fibrous structure lacking cells.

Hematoxylin/eosin (H/E) and orcein staining of PEA resected specimens are shown in Fig. 1A,B. Figure 1C shows an intima layer, expressing endothelial markers, a small compact alpha smooth muscle actin (α-SMA) positive media layer and an enlarged α-SMA negative remodeled intima (neointima) occupying on average 89.2 ± 3.9% of the total width of the specimen (supplementary Table 5). Figure 1D illustrates the presence of mature microvessels within the neointima positive for endothelial markers surrounded by a layer of α-SMA positive cells. The average number of microvessels/mm^2^^2^ per specimen was 47.5 ± 14.2 (supplementary Table 5). The H/E staining of the non-resolved thrombus shows a fibrous structure with no cells (DAPI negative) (Fig. 1E–F). Flow cytometry analysis of disaggregated PEA specimens showed a cellular average of 11.1% CD45^+^, 11.8% α-SMA^+^, 6.7% CD34^+^, 4.9% CD144^+^, 10.9% CD31^+^ among others (n = 20) (supplementary Table 5).

### Isolation and characterization of ECs from PEA material

**Figure 2:**
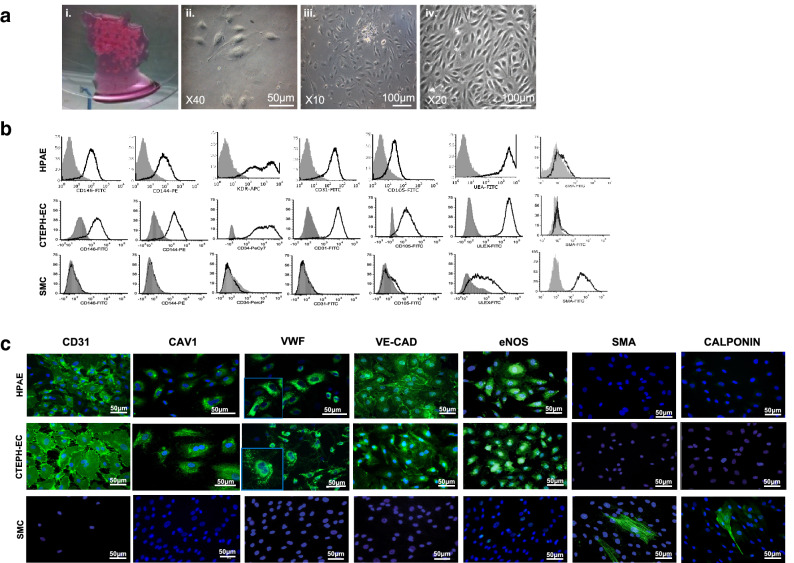
CTEPH-EC characterization. (**a**) Images showing expansion of CTEPH-EC isolated from PEA specimen, (i) minced PEA material, (ii) endothelial colony at day 10, (iii) day 14 and (iv) confluent CTEPH-EC. (**b**) Representative flow cytometry histograms of HPAE, CTEPH-EC and smooth muscle cells (SMC) labelled with antibodies against endothelial surface makers (CD31, UEA-1, vWF, VE-CAD, eNOS) and muscular markers (α-SMA and calponin) (n = 10). (**c**) Representative images of HPAE, CTEPH-EC, and SMC were immune-labelled with antibodies against endothelial makers (CD31, CAV1, vWF, VE-CAD, eNOS) and muscular markers (α-SMA and calponin) (n = 10). Nuclei were counterstained using DAPI (blue) × 40 magnification.

**Figure 3:**
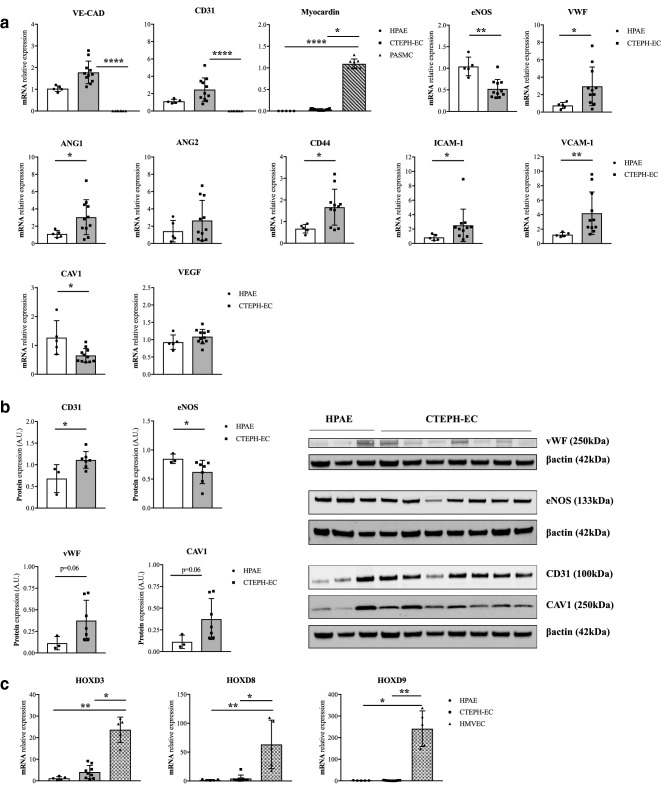
Expression of cell-specific markers in CTEPH-EC. (**a**) CTEPH-EC presented endothelial markers at the mRNA level and were negative for muscular markers (myocardin). Values expressed as mean ± SD, n = 5 HPAE, n = 11 CTEPH-EC and n = 8 SMC independent experiments performed in triplicate, *p* < 0.05*, *p* < 0.01**, *p* < 0.0001****, Mann–Whitney *U* test comparing two groups and Kruskal–Wallis test followed by Dunn’s multiple comparisons test for three groups. (**b**) CD31, eNOS, vWF and Cav-1 protein levels in CTEPH-EC compared to HPAE. Values expressed as mean ± SD, n = 3 HPAE, n = 7 CTEPH-EC independent experiments, *p* < 0.05*, Mann–Whitney *U* test, (full-length gels are presented in supplementary material Fig. 7). The intensity of the individual bands was quantified using freely available Image Lab software (Biorad laboratories), version 6.1.0 build7, http://www.biorad.com. (**c**) CTEPH-EC and HPAE expression levels of HOXD3, -8 and -9 compared to HMVEC-L. Values expressed as mean ± SD, n = 5 HPAE, n = 9 CTEPH-EC and n = 5 HMVEC-L independent experiments performed in triplicate, *p* < 0.05*, *p* < 0.01**, Kruskal–Wallis test followed by Dunn’s multiple comparisons test. Statistical analysis was performed with GraphPad Prism 7 software, version 7.0e, serial number:GP7-0633739-R###-#####, https://www.graphpad.com.

ECs were obtained from fresh PEA resected specimens. Colonies emerged after 7–20 days in culture and continued to proliferate to form a confluent monolayer (Fig. 2A). Efficiency of CTEPH-EC isolation was determined by endothelial colony appearance. CTEPH-EC had cobblestone morphology, typical of ECs (Fig. 2A). CTEPH-EC strongly expressed endothelial surface antigens comparable to HPAE (> 75% for all). This phenotype was maintained throughout cell culture. CTEPH-EC were negative for calponin and α-SMA (< 0.1%) (Fig. 2B,C). Cells also stained positive for endothelial nitric oxide synthase (eNOS) and with localized cytoplasmic granular organelles (Weibel-Palade bodies) (Fig. 2C). CTEPH-EC were confirmed to be ECs at mRNA and protein level (Fig. 3A,B). CTEPH-EC showed an increased expression level of VE-cadherin, CD31, ANG1, vWF, ICAM-1, CD44 and VCAM-1 expression levels compared to HPAE (Fig. 3A,B). No significant differences in membrane permeability were found between CTEPH-EC and HPAE (supplementary Fig. 2). CTEPH-EC showed significant reduction in eNOS expression compared to HPAE (Fig. 3A,B). The levels of VEGF-A and ANG2 were not significantly different between CTEPH-EC and HPAE (Fig. 3A). Additionally, whereas homeobox-(HOX)-containing genes HOXD3, HOXD8 and HOXD9 were highly expressed in HMVEC-L, they were virtually non-expressed in both macrovascular HPAE and CTEPH-EC (Fig. 3C).

### CTEPH-EC showed a hyperproliferative phenotype

**Figure 4:**
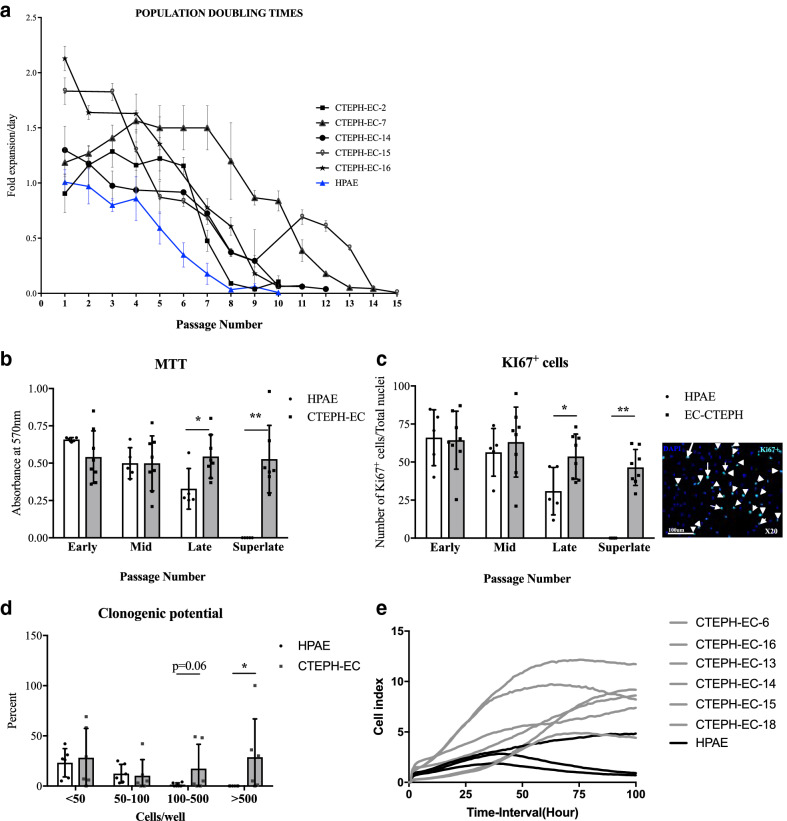
Proliferation capacities of CTEPH-EC. (**a**) Proliferative capacity of CTEPH-EC as population doubling time. n = 3 HPAE, CTEPH-EC, n = 5 independent experiments performed in triplicate (**b**) Viability of CTEPH-EC compared to HPAE evaluated by MTT assay. Values expressed as mean ± SD, n = 5 HPAE, n = 8 CTEPH-EC independent experiments performed in triplicate, *p* < 0.05*, *p* < 0.01**, Mann–Whitney *U* test. (**c**) Quantification and stain of proliferative marker Ki-67 in CTEPH-EC and HPAE at different passages. Values expressed as mean ± SD, n = 5 HPAE, n = 8 CTEPH-EC; independent experiments *p* < 0.05*, *p* < 0.01**, Mann–Whitney *U* test, (**d**) Clonogenic potential of CTEPH-EC expressed in percentage. Values expressed as mean ± SD, n = 6 HPAE, n = 6 CTEPH-EC; independent experiments, *p* < 0.05*, Mann–Whitney *U* test. (**E**) Cellular adhesion of CTEPH-EC compared to HPAE. n = 3 HPAE, n = 6 CTEPH-EC. Statistical analysis was performed with GraphPad Prism 7 software, version 7.0e, serial number:GP7-0633739-R###-#####, https://www.graphpad.com.

Proliferative capacity was assessed by quantifying the fold cell expansion/day. Growth of CTEPH-EC was consistently enhanced and showed a greater area under the curve compared to HPAE (9.6 ± 2.5 *vs* 4.4 ± 0.2 respectively) (Fig. 4A). CTEPH-EC grew for > 10 passages *versus* HPAE that lost growth potential around passage 10 (Fig. 4A). CTEPH-EC showed a significantly longer viability at late passages (Fig. 4B). CTEPH-EC had increased number of Ki-67^+^ cells (Fig. 4C), higher clonogenic potential (Fig. 4D), and greater cell proliferation compared to HPAE at late passages (Fig. 4E).

### CTEPH-EC showed resistance to apoptosis

**Figure 5:**
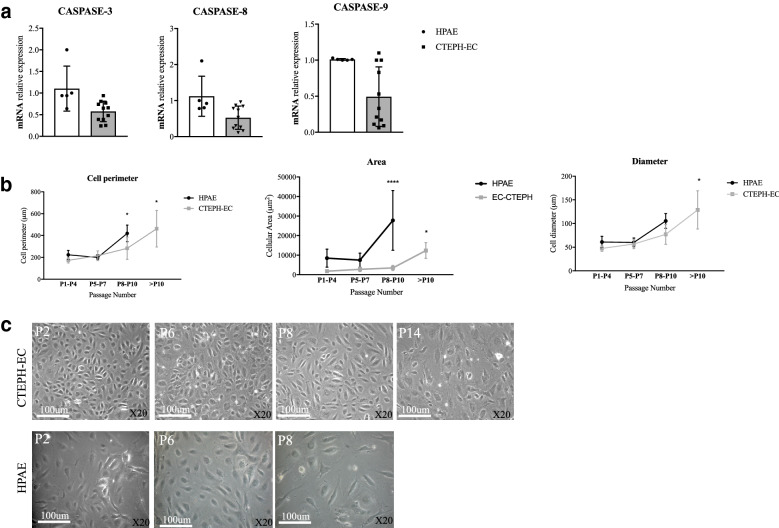
Apoptosis in CTEPH-EC. (**a**) mRNA expression profile of apoptotic markers caspase-3, -8 and -9 in CTEPH-EC compared to HPAE. Values expressed as mean ± SD, n = 5 HPAE, n = 11 CTEPH-EC independent experiments performed in triplicate, *p* < 0.05*, Mann–Whitney *U* test. (**b**) Cellular perimeter, area, and diameter of CTEPH-EC and HPAE measured at different passages. Values expressed as mean ± SD, n = 8 HPAE, n = 8 CTEPH-EC independent experiments performed in triplicate. *p* < 0.05*, *p* < 0.0001****, two-way ANOVA followed by Tukey´s multiple comparisons test. (**c**) Representative images of CTEPH-EC and HPAE cells at different cell passages. Cellular circumference, area and diameter were measured in triplicate fields of 5–10 cells/picture (20 × magnification) using freely available imaging processing ImageJ software, version:2.1.0/1.53c, http://imagej.net/contributors. Statistical analysis was performed with GraphPad Prism 7 software, version 7.0e, serial number:GP7-0633739-R###-#####, https://www.graphpad.com**.**

Compared to HPAE, CTEPH-EC showed a significant reduction of caspase-3, -8, -9 expression levels (Fig. 5A). mRNA expression levels of BCL2, p53 and p21 remained unchanged (supplementary Fig. 3). Cellular circumference, area, and diameter of CTEPH-EC at late passage was reduced compared to HPAE, consistent with a more immature and proliferative cellular phenotype (Fig. 5B,C).

### CTEPH-EC showed reduced angiogenic capacity

**Figure 6:**
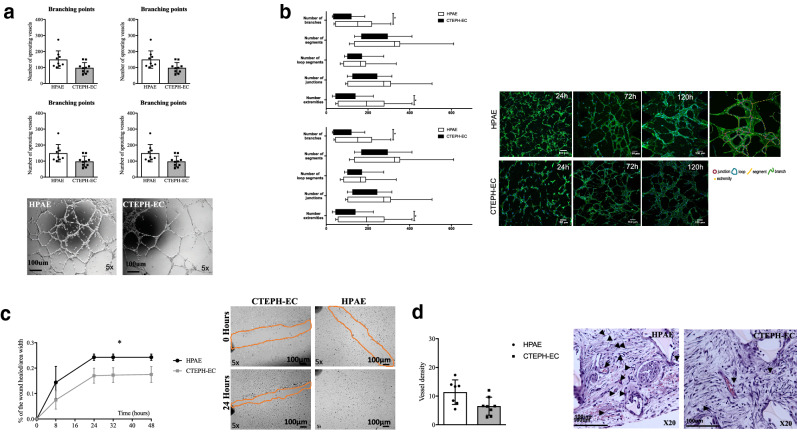
Functional characterization of CTEPH-EC. (**a**) In vitro angiogenic potential of CTEPH-EC quantified by the number of branching points, tube length, cell covered area and number of loops. Representative pictures of spontaneous tube formation in HPAE and CTEPH-EC, n = 9 HPAE, n = 10 CTEPH-EC independent experiments performed in triplicate, *p* < 0.05*, *p* < 0.01**, Mann–Whitney *U* test, values expressed as mean ± SD. (**b**) Quantification of cell connections and geometrical features measured at 24, 72 and 120 h and representative pictures of HPAE and CTEPH-EC. *p* < 0.05*, *p* < 0.05*, Mann–Whitney *U* test values expressed as mean ± SD. (**c**) Migration capacity of CTEPH-EC and HPAE in the wound healing assay. Percentage of wound closure is measured at 8, 24, 32 and 48 h and plotted as the percentage of closure over the average area of width. Representative pictures of wound closure at 0 h and 24 h in HPAE and CTEPH-EC, n = 6 HPAE, n = 7 CTEPH-EC, *p* < 0.01**, Mann–Whitney *U* test at 8 h, 24 h, 32 h and 48 h, values expressed as mean ± SD. (**d**) In vivo angiogenic potential of CTEPH-EC- and HPAE-embedded sponge pellets quantified by the number of vessels in the sponge sections. Representative pictures of vessels in sponges embedded with HPAE and CTEPH-EC. n = 7 HPAE, n = 8 CTEPH-EC independent experiments, *p* < 0.05*, Mann–Whitney *U* test, values expressed as mean ± SD. Statistical analysis was performed with GraphPad Prism 7 software, version 7.0e, serial number:GP7-0633739-R###-#####, https://www.graphpad.com.

Both CTEPH-EC and HPAE formed tube-like structures on a Matrigel and on 3D fibrin hydrogel membrane matrix (Fig. 6A,B, and supplementary video 1–2). Quantification of cell–cell connections and tube length showed reduced angiogenic potential of CTEPH-EC compared to HPAE (Fig. 6A,B). CTEPH-EC also displayed reduced recovery capacity in wound healing assay (Fig. 6C). In vivo, we examined the spontaneous vascularization of subcutaneously implanted sponge embedded with CTEPH-EC and HPAE. Quantification of number of vessels showed a significant reduction in growth of new vessels in CTEPH-EC loaded sponges compared to HPAE (Fig. 6D). There was no significant difference in spontaneous vascularization between control sponges (Matrigel only) and sponges with CTEPH-EC (5.8 ± 2.3 vs 6.48 ± 3.1 respectively). The expression levels of Notch related genes/proteins did not differ between CTEPH-EC and control cells (supplementary Fig. 4).

### Mitochondrial abnormalities in CTEPH-EC

**Figure 7:**
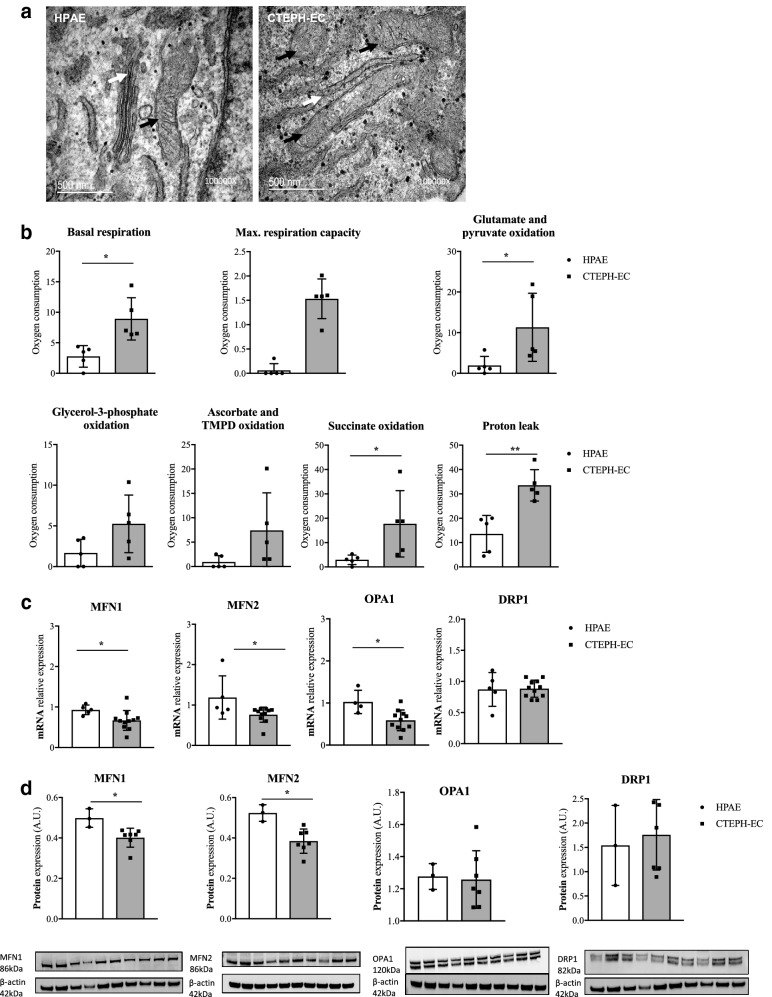
Mitochondria in CTEPH-EC. (**a**) Visualization of the mitochondrial structure of cultured CTEPH-EC and HPAE by electron microscopy. (**b**) Oxygen consumption was measured in CTEPH-EC and HPAE by high-resolution respirometry (Oroboros Instruments). Contribution of the individual complexes of the mitochondrial respiratory chain to total cellular respiration was determined by the use of substrates or inhibitors of specific mitochondrial respiratory chain complexes. At last, proton leak was measured in CTEPH-EC and HPAE. Values expressed as mean ± SD, n = 5 HPAE, n = 5 CTEPH-EC independent experiments, *p* < 0.05*, *p* < 0.01**, Mann–Whitney *U* test. (**c**, **d**) mRNA expression and protein expression of fusion (MFN1, MFN2, and OPA1) and fission (DRP1) related modulators in CTEPH-EC and HPAE. n = 5 HPAE, n = 11 CTEPH-EC independent experiments performed in triplicate for mRNA expression for MFN1, MFN2, DRP1; and n = 4 HPAE, n = 11 CTEPH-EC independent experiments performed in triplicate for mRNA expression for OPA1. For western blot experiments, values expressed as mean ± SD, n = 3 HPAE, n = 7 CTEPH-EC, *p* < 0.05*, Mann–Whitney *U* test, (full-length gels are presented in supplementary material Fig. 8). The intensity of the individual bands was quantified using freely available Image Lab software (Biorad laboratories), version 6.1.0 build7, http://www.biorad.com. Statistical analysis was performed with GraphPad Prism 7 software, version 7.0e, serial number:GP7-0633739-R###-#####, https://www.graphpad.com.

Electron microscopy of cultured CTEPH-EC showed irregular mitochondrial structure (inner membranes and cristae) compared to healthy HPAE (Fig. 7A). The number of mitochondria per cell area and mitochondria circularity did not differ between CTEPH-EC and HPAE (supplementary Fig. 5A,B). Aspect ratio or mitochondrial elongation parameters were also comparable between the two groups (supplementary Fig. 5C). Mitochondrial content assessed by MitoTracker green and by mt12SrRNA gene/nRNAseP nuclear gene ratio did not differ between the two groups (supplementary Fig. 5D,E).

Oxygen consumption, measured by high resolution respirometry, was increased in CTEPH-EC compared to HPAE. Both endogenous cell respiration (basal), as well as maximum respiratory capacity were increased (Fig. 7B). To explore whether increased basal and maximum respiration were related to a specific complex of the mitochondrial respiratory chain (MRC), we further explored each MRC complex through stimulation or inhibition with specific substrates. All oxidative activities were increased in CTEPH-EC compared to control cells, reaching statistical significance for complex-I and-II (Fig. 7B). Finally, significant increase in proton leakage was observed in CTEPH-EC suggesting uncoupling leakage as the main causative factor for the oxidative alterations (Fig. 7B).

### CTEPH mitochondrial fusion/fission

Mitochondrial dynamics in HPAE and CTEPH-EC were analyzed by studying both fusion and fission processes. All fusion genes studied (MFN1, MFN2 and OPA1) showed a significant downregulation in CTEPH-EC compared to HPAE (Fig. 7C,D). Fission related gene DRP1 remained unchanged (Fig. 7C,D).

### CTEPH-EC showed high levels of oxidative stress

**Figure 8:**
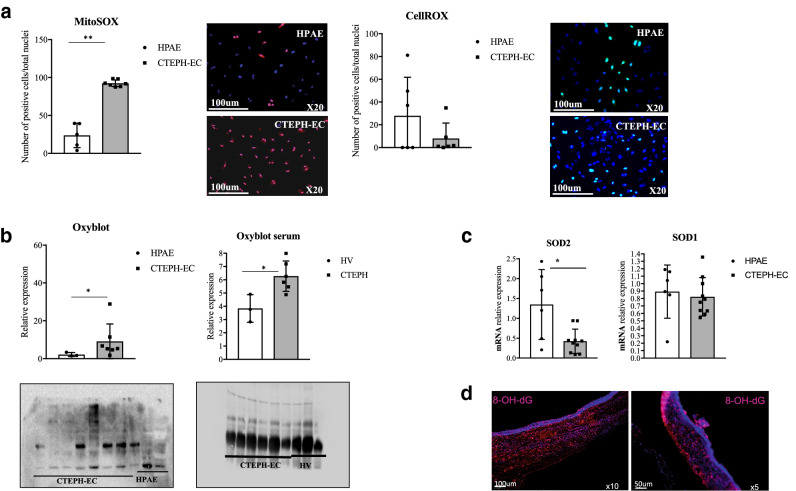
Oxidative stress in CTEPH-EC. (**a**) ROS levels in CTEPH-EC and HPAE were visualized by fluorogenic dye MitoSOX (red) n = 5 HPAE, n = 7 CTEPH-EC independent experiments, *p* < 0.01**, Mann–Whitney *U* test, values expressed as mean ± SD and CellROX (green) n = 6 HPAE, n = 6 CTEPH-EC, *p* > 0.05. (**b**) Oxidation status of proteins in CTEPH-EC, HPAE, patient serum and serum from healthy volunteers. n = 3 HPAE, n = 7 CTEPH-EC; n = 3 HPAE, n = 6 HV, *p* < 0.05*, Mann–Whitney *U* test, values expressed as mean ± SD. The intensity of the bands was quantified using Image Lab software (Biorad laboratories), version 6.1.0 build7, http://www.biorad.com. (**c**) mRNA expression of SOD1 and SOD2 in CTEPH-EC and HPAE. Values expressed as mean ± SD, n = 6 HPAE, n = 10 CTEPH-EC independent experiments performed in triplicate, *p* < 0.05*, Mann–Whitney *U* test. (**d**) Staining of DNA damage induced by oxidative stress in PEA specimen using 8-hydroxy-2′-deoxyguanosine (8-OHdG). Statistical analysis was performed with GraphPad Prism 7 software, version 7.0e, serial number:GP7-0633739-R###-#####, https://www.graphpad.com.

Whereas direct measurement of total ROS levels showed no difference between the two cell lines there was a significant increase of mitochondrial reactive oxygen species (mROS) production in CTEPH-EC compared to HPAE (Fig. 8A). Oxyblot assay for immunodetection of carbonyl groups showed a significant upregulation of oxidized proteins in CTEPH-EC compared to HPAE (Fig. 8B). Increased levels of oxidized proteins were also seen in serum of CTEPH patients compared to healthy controls (Fig. 8B). Superoxide dismutase-2 (SOD2) expression was significantly reduced in CTEPH-EC compared to HPAE (Fig. 8C). Superoxide dismutase-1 (SOD1) expression levels showed no significant difference between the two groups (Fig. 8C). Additionally, levels of 8-hydroxyguanosine (8-OHdG), a biomarker of DNA damage, were abundant in PEA samples (Fig. 8D).

### CTEPH-EC correlation with clinical data

Patient’s characteristics are summarized in supplementary Table 1. CTEPH patients studied had a mean age of 62.5 ± 6.5 years old and gender matched. Lower CTEPH-EC eNOS mRNA levels were observed in patients classified in worst World Health Organization functional classification (WHO-FC) (supplementary Fig. 6). No other dysfunctional characteristics found in CTEPH-EC were significantly related with clinical risk parameters.

## Discussion

In this study, we isolated endothelial cell lines from material collected at PEA and generated an in vitro model of endothelial pathology in CTEPH. CTEPH-EC presented an abnormal hyperproliferative phenotype, a reduced angiogenic capacity and eNOS expression, an overexpression of adhesion molecules and a dysregulated mitochondrial function with an enhanced oxidative stress.

### Isolation and characterization of CTEPH-EC

CTEPH-EC showed typical cobblestone morphology and stained positive for a wide range of endothelial markers and negative for muscular or hematopoietic markers. Homeobox (HOX) genes are well conserved among mammalian species and good predictors of EC identity^18^. Toshner et al. identified a differential expression of certain HOX genes between lung microvascular ECs and pulmonary artery macrovascular ECs^18^. Our results indicate that isolated CTEPH-EC were negative for the microvascular HOX-containing genic pattern suggesting that these patient endothelial cells were derived from the local pulmonary artery endothelium and not from newly infiltrating neovessels found within PEA material. This is important as abnormalities outlined in this study specifically refer to the damaged proximal pulmonary artery endothelium and allow us to further understand the involvement of patient´s pulmonary artery endothelial cells in vessel obstruction and remodeling.

It has been recently shown that ECs derived from patients with PAH (PAH-EC) have an hyperproliferative and an apoptosis-resistant phenotype contributing to the progression of the disease^19^. In this study, we showed that CTEPH-EC also grew at a consistently faster rate and presented higher number of Ki-67^+^ cells when compared to healthy endothelial cells. This result is in line with previous publications in which they also showed greater proliferation capacity of CTEPH-EC denoting the involvement of endothelial cells in pulmonary artery occluding lesions^10,20^. In this study, we additionally demonstrated that CTEPH-EC had a significantly higher clonogenic potential and could survive for many more cell culture passages without losing their proliferative potential and phenotype. As reported for PAH-EC, CTEPH-EC also presented a significant decrease in apoptotic caspase related genes compared to controls. However, the expression of survival factors such as BCL2 or tumorigenic-associated genes p53 and p21 did not differ between CTEH-EC patient and control cells. Additionally, CTEPH-EC maintained a smaller cell size throughout cell culture passages consistent with a less differentiated phenotype. Altogether, these results strongly support that CTEPH-EC present a more viable, proliferative phenotype compared to pulmonary ECs derived from healthy subjects. Clinically, these results are relevant as a decontrolled endothelial cell proliferation is likely to contribute to the development of occluding lesions and eventually vessel remodeling, in non-occluded arteries hence contributing to increased pulmonary vascular resistance. Indeed, Mercier et al., have shown that CTEPH-EC cell culture medium is able to enhance pulmonary artery smooth muscle cell growth and monocyte migration^11^. These results emphasize the need to understand CTEPH endothelial cell pathology to find novel treatments to reverse such dysfunction and to prevent neighboring cell transformation.

### CTEPH-EC overexpression of adhesion molecules

CTEPH-EC showed a significant increase in expression of adhesion molecules (CD31, VCAM-1, ICAM.1, CD44) compared to healthy HPAE cells. This result is in line with a previous study showing higher plasma levels of adhesion molecules (VCAM-1 and ICAM-1) compared to controls^11^. Several studies have shown the role of CD31 in modulating apoptosis and cell growth under stress conditions^21^. Cheung et al. reported that in response to apoptotic stimuli, CD31 engages a pro-survival pathway that in some cancers influences tumor immuno-resistance^22^. Tsuneki et al. showed that CD44 could regulate endothelial proliferation and apoptosis by modulating the expression of CD31 and VE-cadherin^23^. The increased expression of adhesion molecules in CTEPH-EC suggests a possible correlation with CTEPH-EC pro-survival characteristics. It has been shown that CTEPH patients presented higher plasma levels of circulating C-reactive protein (CRP) than controls^24^. Additionally, our recent results also identify a basal inflammatory profile of CTEPH-EC^25^. Both studies highlight the important role of NF-kB signaling pathway and CRP in increasing the expression of ICAM-1 and cell adhesion capacity in CTEPH-EC^26,27^. Our results did not show an increase in endothelial permeability in CTEPH-EC compared to controls and further connections between cellular inflammation, permeability and cellular adhesion molecules deserve further investigation. Overall, these studies indicate the importance of inflammation and adhesion molecules to understand the mechanisms behind the disease. Targeting adhesion molecules as early indicators of endothelial dysfunction deserves further study to restore endothelial function in CTEPH.

### CTEPH-EC function

Functionally, CTEPH-EC had reduced capacity to form tube-like structures and decreased migration potential in response to wounding. In agreement with this, previous studies reported an impairment of angiogenesis in CTEPH patients. Alias et al*.,* showed a downregulation of mRNA levels of VEGFR2 in PEA specimens^28^. Also, Zabini et al. reported that homogenized PEA specimens contained several cytokines that inhibit angiogenesis^29^. We comprehensively assessed angiogenesis, demonstrating that the formation of tubular structures by CTEPH-EC is functionally impaired both in vitro and in vivo. Whereas HPAE cells showed a pro-angiogenic effect promoting spontaneous vascularization in vivo, CTEPH-EC did not. This result indicates that CTEPH-EC and its secretome did not effectively enhance vessel formation, in line with previous outlined results^28,29^. However, in our series VEGF mRNA levels did not differ between CTEPH-EC and controls. Larger studies measuring plasma levels of VEGF and other angiogenic related cytokines will be of interest. Additionally, our results also demonstrate reduced cellular migration in CTEPH-EC compared to control cells. CD31 overexpression has been shown to inhibit migration of ECs through a PECAM-1/γ-catenin/desmoplakin/vimentin dependent mechanism^21^. Accordingly, overexpression of adhesion molecules in CTEPH-EC could impair their angiogenic potential and cellular migration. Our results show a disbalance between EC proliferation and differentiation as isolated CTEPH-EC appear to be more determined to increase their mitogenic activity and viability rather than to promote angiogenesis or migration. New therapies should target to correct this disbalance and aim to re-establish the proliferation/differentiation equilibrium.

### Mitochondrial dysfunction in CTEPH-EC

Endothelial metabolism is closely linked to EC function^30^. Accordingly, a better understanding of the metabolic changes in CTEPH-EC is a crucial step in resolving CTEPH pathogenesis. It has been shown that PAH-EC have an altered mitochondrial-metabolic phenotype with a metabolic shift towards glycolysis and an increased mitochondrial fusion/fission imbalance^31^. Compounds stimulating TCA cycle are under investigation for PAH treatment^32^. The question is whether such molecules could also be applied to CTEPH patients. Our results show that fusion regulatory GTPases MFN1, MFN2 and OPA1 were downregulated in CTEPH-EC compared to control cells. However, unlike in PAH-EC, fission regulatory protein DRP1 was not changed. Downregulation of MFN2 in CTEPH-EC indicates mitochondrial unbalance and damage.

On the other hand, our electron microscopy results showed abnormal mitochondria with irregular inner membrane and cristae in CTEPH-EC. Cristae shape determines the assembly and stability of respiratory chain super-complexes, impacting mitochondrial respiratory efficiency^33^. In our study, CTEPH-EC displayed increased mitochondrial respiration activity in all the respiratory chain complexes. Increased oxygen consumption could be due to a rise of mitochondrial mass or due to proton leakage. As we did not find an increase in number of mitochondria, we imply the observed uncoupling leakage as main causative factor for oxidative alterations in CTEPH-EC. In agreement with these results, we confirmed the presence of higher levels of mitochondrial reactive oxygen species (mROS) production in CTEPH-EC. Taken together, our results show that CTEPH-EC mitochondria are dysfunctional with a fusion/fission imbalance, an increased mitochondrial respiration and uncoupling leakage. Sustained abnormal mitochondria dynamics in CTEPH-EC could trigger important deficits in cell bioenergetics, mitochondrial function and increased mROS production which at the same time faces a significant threat to neighboring tissue. It is not known whether the endothelial hyperproliferative and hypoangiogenic phenotype described above, could be the cause or consequence of altered mitochondrial dynamics. However, altogether leads to a vicious cycle that potentially contributes to the endurance of endothelial dysfunction and hemodynamic impairment. Further studies are needed to understand the causality and the impact of mitochondrial dysfunction in the pathogenesis of CTEPH that eventually could open a new therapeutic approach with mitochondria-specific therapies. Additionally, the mitochondrial dysfunction abnormalities described here are significantly different from the mitochondrial imbalance previously reported in PAH-EC^31^. This uncovers differences in mitochondrial dynamics between PAH and CTEPH that should be taken into account for future therapeutic strategies^31^.

### Oxidative stress

Higher levels of protein carbonyl groups and 8-OHdG, indicators for the oxidative status of proteins and DNA damage, were observed in CTEPH-EC in comparison to healthy ECs. Oxidative stress is caused by elevated ROS production and/or reduced cellular detoxification^34^. Antioxidant enzyme SOD2 is a first line of defense against superoxide accumulation and cell death^34,35^. In PAH levels of SOD2 are downregulated causing ionic dysregulation and downstream pulmonary vasoconstriction^36^. Our results show that SOD2 mRNA levels were also significantly downregulated in CTEPH-EC. Long-term excessive free radical production in CTEPH-EC released by mitochondria through metabolic processes, might constitute an essential element to the chronification of the disease and maintenance of the endothelial dysfunction vicious cycle. This imbalance might represent a potential target to reverse vascular remodeling and disease progression in CTEPH. Epigenetic attenuation of SOD2 could be one of the keys behind CTEPH-EC dysfunctionalities.

Decreased eNOS activity has also been associated with mitochondrial impairment and dysregulated angiogenesis in PH^37^. In agreement with these findings, our study showed that increased levels of mROS in CTEPH-EC were accompanied by a significant reduction in eNOS and caveolin-1 expression. Previous studies highlighted the importance of eNOS for the correct functioning of endothelial cells^38^. Reduced angiogenesis, mitochondrial dysfunction and cell migration abnormalities found in this study could be linked to an eNOS downregulation. Understanding the underlying signaling in oxidative/nitrative stress-induced pathways in CTEPH, merits further attention^39^. This study clearly points out that CTEPH-EC have an abnormal behavior with many endothelial cell facets disturbed. It is necessary to further investigate the links between them to ultimately envisage the optimal strategy to correct such endothelial disbalance.

### Correlation with clinical features

Lower CTEPH-EC eNOS mRNA levels were seen in patients with worst WHO-functional class and merits further investigation as potential predictive and diagnostic tool in CTEPH. Previous studies have shown that impaired neovascularization, defined as the number of neovessels present in the PEA neointima, was associated to an adverse clinical outcome and that the levels of circulating VEGF inversely correlated with mPAP measured 3 days after PEA^13^. However, in our series, the levels of VEGF mRNA from CTEPH-EC isolated from PEA tissues or the number of EC-CTEPH tubule structures in vitro did not correlate with any clinical parameter analyzed. Identification of an impaired neoangiogenesis and quantification of the levels of angiogenic growth factors in future CTEPH studies would be of particular interest.

### Limitations

The use of primary cell cultures and the isolation method used in this study could involve involuntary selection of cells with the highest growth potential. To minimize this limitation, all CTEPH-EC were isolated strictly following the same protocol and all experiments were carried out in early passage cells (unless otherwise stated). We confirm that CTEPH-EC derived from material collected at PEA are dysfunctional. However, it is not possible to conclude that functional differences found in CTEPH-EC are a primary cause or a secondary consequence of the haemodynamics perturbations caused by the pulmonary vascular occlusions. Further studies would need to be designed to answer this specific subject. All CTEPH-EC were derived from patients undergoing PEA and therefore representing CTEPH patients with advanced stages of the disease suitable for surgical treatment. Patients with more distal vascular occlusions non amenable with PEA may show different EC behavior. Additionally, the number of patients used to correlate with CTEPH-EC characteristics is rather small and larger cohort studies are needed to confirm these findings. Finally, healthy individuals cannot undergo to a pulmonary endarterectomy procedure as CTEPH patients. Endothelial cells between patients and controls are both originated from human pulmonary arteries and the isolation and cell culture techniques are essentially the same, even though, the starting material to isolate endothelial cells is not identical.

## Conclusions, future perspectives, and clinical relevance

Our study represents the largest comprehensive investigation of isolated CTEPH-EC. We developed an in vitro CTEPH-derived endothelial cell model to evaluate its endothelial status and we showed that endothelial function is disturbed and might play a crucial role in the pathogenesis of CTEPH. CTEPH-EC isolated from PEA specimens present marked dysfunctionalities in vitro and in vivo. We have identified several novel molecular pathways likely to influence thrombus chronification, vessel wall remodeling and development of CTEPH. Understanding the molecular fingerprint of endothelial dysfunction in CTEPH offers opportunities to find new therapeutic targets and biomarkers to develop an effective personalized disease management and increase life-expectancy.

## Supplementary Information

Supplementary Video 1.

Supplementary Video 2.

Supplementary Information 1.

## Data Availability

All data relevant to the study are included in the article or uploaded as supplementary information.
