# Supplementary material for: Derivation and characterisation of endothelial cells from patients with chronic thromboembolic pulmonary hypertension
Source: Sci Rep. 2021 Sep 22;11:18797. doi: 10.1038/s41598-021-98320-1 (PMC8458486; doi:10.1038/s41598-021-98320-1)
Supplement: Supplementary file 3 — Supplementary Information 1. [file 41598_2021_98320_MOESM3_ESM.pdf]

Supplementary Material
DERIVATION AND CHARACTERISATION OF ENDOTHELIAL CELLS FROM PATIENTS WITH CHRONIC THROMBOEMBOLIC PULMONARY HYPERTENSION.
.

Olga Tura-Ceide*+[1,2,3], Valérie F.E.D. Smolders*[1,4,5], Núria Aventin[1], Constanza Morén[6,7], Mariona Guitart-Mampel[6], Isabel Blanco[1,2], Lucilla Piccari[1], Jeisson Osorio[1], Cristina Rodríguez[1,3], Montserrat Rigol[8,9], Núria Solanes[8], Andrea Malandrino[10], Kondababu Kurakula[11], Marie Jose Goumans[11], Paul H.A. Quax[5], Victor I. Peinado[1,2], Manuel Castellà[12], Joan Albert Barberà+[1,2]

*Both authors contributed equally to the work

+Correspondence: olgaturac@gmail.com and jbarbera@clinic.cat. Servei de Pneumologia, Hospital Clínic. Villarroel, 170. Barcelona 08036. Spain. Phone 34-93-227-5747; Fax. 34-93-227-5455.

Word count: 3089
Running title: Endothelial dysfunction in CTEPH
Table of contents: Detailed methodology and supplementary Tables and Figures.

DETAILED MATERIALS AND METHODS

Subjects

Fourteen subjects with CTEPH, aged between 55-75 years (53.3% male) who underwent PEA at the Hospital Clinic of Barcelona, Spain were enrolled in the study. Patient characteristics are shown in Table 1. CTEPH was diagnosed according to current guidelines [15]. The study was conducted in accordance with the Declaration of Helsinki, approved by the institutional Committee on Human Research (Hospital Clínic of Barcelona ethics committee (HCB/2018/0837 and HCB/2018/0434) and all subjects gave written informed consent.

Morphometric and histological assessments

A piece of PEA resected material from the vessel wall accessible to surgery was fixed in 4% paraformaldehyde (PFA) and paraffin embedded. Sections (5μm) were stained by hematoxylin and eosin (H/E; nuclei and cytoplasm) and orcein (elastin fibers). Staining of elastin was performed using orcein stain. The thickness of neointima (remodeled intima) was measured as the distance from lumen to the media layer. Microvessel density within the neointima was assessed as the number of blood vessels per mm^2/tissue. Distribution of endothelial markers (vWF and CD31), vascular smooth muscle cell markers (α-SMA) and 8-Hydroxyguanosine (8-OH-dG) was analyzed by immunohistochemistry. Immunohistofluorescence staining was performed as previously described [16]. Briefly, paraffin sections were deparaffinized, rehydrated. and boiled for 40 min in Vector Antigen Unmasking Solution (Vector) using a pressure cooker. After blocking with 1% BSA in 0.1% Tween-PBS, sections were incubated overnight at 4°C with primary antibodies directed against alpha smooth muscle actin (SMA; 1:5000; Sigma), Von Willebrand factor (1:1000, Abcam), CD31 (1:1000; Santa Cruz) and 8-OHdG (1:150; Bioss Antibodies). All sections were mounted with ProLong Gold antifade reagent (Invitrogen) containing DAPI. In parallel, another piece of PEA resected material from the vessel wall accessible to surgery was enzymatically digested using collagenase type I and IV and incubated 60 min a 37°C. The digested sample was directly stained and analyzed by flow cytometry for phenotypic expression of surface markers using pre-conjugated anti-human monoclonal antibodies (mAbs), as previously described [39]. The antibodies used are listed in supplementary Table 2.

Primary cell cultures

Isolated ECs (CTEPH-EC) from all n=14 subjects were obtained from fresh PEA resected specimens by mincing it into 1-2 mm pieces and culturing in 0.2% gelatin-coated plates in EBM-2 EC growth medium (Lonza), supplemented with 10% fetal bovine serum (FBS) and EGM-2 SingleQuots (Lonza). Cell colonies appeared after 7-20 days in culture. Cells were used between passages 1 and 15 (proliferation, viability and senescence assays) and between passages 4 and 7 (for all other assays). Passages <4 are referred to as early, passages between 4 and 7 are referred to as mid, passages between 7 and 10 are referred to as late and passages >10 are referred to as super-late. All cellular experiments were performed at a cell confluency of 80-90% unless otherwise stated. Human pulmonary artery SMCs, human lung microvascular ECs (HMVEC-L) and human pulmonary artery ECs (HPAE) were purchased from Lonza and used as controls. This study included age and gender matched HPAE cells from three independent donors purchased from Lonza (average HPAE age: 54.3±17,6 and 66% male percentage). Control lines were used at passages one to eight and maintained in a humidified atmosphere at 37ºC in 5% CO_2.

Cell characterisation
Cells were directly analyzed by flow cytometry for phenotypic expression of surface markers using pre-conjugated mAbs, as previously described [39]. The anti-human mAbs used included endothelial markers (CD144, KDR, CD34, CD31, CD105, UEA-1 and CD146), hematopoietic lineage markers (CD45, CD14, CD16, CD56, CD62, CD133 and c-Kit), vascular smooth muscle marker α-SMA and annexin V apoptotic marker. Immunofluorescence analysis for cell phenotype was performed as previously described [39] using antibodies against endothelial markers (CD31, UEA-1, vWF, VE-CAD and eNOS) and muscular markers (α-SMA and calponin). The antibodies used are listed in supplementary Table 2. An antibody against Ki-67 (Novocastra) was used to measure cell proliferation.

RNA Isolation and quantitative Real Time PCR
Total RNA was extracted from 80-90% confluent cultures using 1ml of TRIsure reagent (Bioline) according to the manufacturer instructions. Following reverse transcription (high-capacity cDNA RT kit, Applied Biosystems), quantitative real-time PCR experiments were performed in the presence of fluorescent dye (power SYBR Green, Applied Biosystems) with a ViiA 7 Real-Time PCR System (Applied Biosystems). cDNA copy numbers were normalized against genomic DNA level of endogenous β-actin and analyzed by the 2-ΔΔCt method. All primers were delivered by IDT and primer sequences are listed in supplementary Table 3.

Western blotting
Protein was isolated from cells at 80-90% confluency. Protein was isolated using RIPA lysis and extraction buffer (Pierce) supplemented with Halt protease/phosphatase inhibitor cocktail (ThermoFisher Scientific). Protein concentrations were determined using BCA protein assay kit (Pierce) following manufacturer's instructions. Samples were prepared to load 15-25 μg of protein into wells of commercial NuPAGE 4-12% Bis-Tris Gels (Thermo Fisher) alongside Kaleidoscope Precision Plus Protein Standard (BioRad). As loading buffer, NuPAGE LDS Sample Buffer 4X (Thermo Fisher) was used at 1X; and the NuPAGE MES SDS Running Buffer 20X (Life Technologies) diluted to 1X was used as running buffer. Samples were heated at 70ºC for 10 min before loading into the gel for an electrophoresis duration of about 50 min at 200V followed by transfer onto nitrocellulose membrane using the iBlot Gel Transfer Stacks Nitrocellulose, Regular Kit (Thermo Fisher) and iBlot Gel Transfer Device, (Invitrogen), following manufacturer's guidelines. After the transfer process, the membrane was blocked for 1 hour in 1X blocking solution using Casein Blocking Solution 10X (Sigma). Membranes were incubated overnight at 4ºC under rotation in 0.5X Casein Blocking Solution with primary antibodies following the manufacturer recommendations. Antibodies used are listed in supplementary Table 2. The intensity of the individual bands was quantified using freely available Image Lab software (Bio-Rad Laboratories), version 6.1.0 build7, http://www.biorad.com All results are shown as relative expression to β-actin protein levels.

Cell Growth Kinetics
CTEPH-EC and HPAE were plated in triplicate at a concentration of $3x10^4$ cells/ml. At 80% confluence cells were dissociated from the plate by trypsinization and counted. Cells were re-plated in triplicate at the same concentration and passaged until no growth was observed. Proliferative capacity was assessed by quantifying the fold cell expansion/day

as number of final cells divided by the number of seeded cells/days of culture.

Cell Viability

The viability potential of cells through different passages, was determined using Vybrant MTT Cell Proliferation Assay Kit, (Thermo Fisher). Cells at different passages were plated at a density of $2x10^4$ cells per well on a 96-well microtiter plate in EGM-2 medium in a final volume of 100μl. Two wells of 100μl of EGM-2 medium without cells were used as blanks. WST-1/ECS solution was added at 10μl per well, incubated for 4 hours at 37ºC and quantified using multiwell spectrophotometer to measure absorbance of the dye solution at 570 nm.

Single Cell clonogenic assay

Single CTEPH-EC and HPAE were plated in a 96-well plate and cultured as previously described [17]. for 14 days changing media every 4 days. The number of cells per well was counted by visual inspection and classified into four different categories: 2–50 cells/well, 50–500 cells/well, 100–500 cells/well, >500 cells/well.

Cell growth and proliferation assay using xCELLigence

Experiments were carried out using the xCELLigence RTCA DP instrument (Roche Diagnostics) in a humidified incubator at 37ºC and 5% CO_2. 100 mL of cell-free growth medium (10% FBS) was added to the wells and the background impedance for each well was measured. Cells were seeded in parallel into 0.2 % gelatin coated wells at 5,000 cells/well in 150ul medium/well. After leaving the plates at RT for 30 min to allow cell attachment, in accordance with the manufacturer's guidelines, they were loaded into the RTCA DP device in the incubator. Impedance value of each well was monitored by the xCELLigence system and expressed as a Cell Index value (CI). The CI represents the measure of cellular adhesion across each individual well. In the absence of living cells, the CI values will be close to zero. After cellular attachment onto the electrode, the measured signal correlates linearly with cell number throughout the experiment [40]. Cells were incubated for 5 days in EGM-2 growth medium (10% FBS) and CI was monitored every 5-15 min.

Cell Morphology

Cellular circumference, area and diameter were measured using freely available imaging processing ImageJ software, version:2.1.0/1.53c, http://imagej.net/contributors in triplicates fields of 5-10 cells/picture (20x magnification).

Tube formation assay

10µL of Matrigel (BD Biosciences) was added to each well of an ibiTreat µ-Slide Angiogenesis, (Ibidi) and allowed to polymerize for a minimum of 30 min at 37°C. EC lines were resuspended in EC medium and seeded in each well at a concentration of 1×10^4 cells/well in a 50µl total volume. Cells were monitored to determine the formation of tube-like structures and pictures (5x) were taken at baseline and at 16h. HPAE were used as a positive control–forming capillary-like structures. Number of branching points, tube lengths, cell covered area and number of loops were quantified in triplicate for CTEPH-EC and HPAE in 5 random fields.

3D microvascular networks (µVN) were obtained by a microfluidic approach [41]. Microfluidic chips were fabricated in house using standard soft-lithography techniques, from a SU-8 master with micro-features using polydimethylsiloxane (PDMS) [41]. The master design included three channels for injection of a mixture of ECM-like fibrin

hydrogel and cells, flanked by four channels injected with culture media. All channels in the chip were 100µm thick, allowing for 3D culture. CTEPH-EC and HPAE were injected at a seeding density of 6-9×10^6 cells/ml and suspended in fibrin in one of the three gel channels. Human lung fibroblasts (HLF, Lonza) were suspended in fibrin and injected in the remaining two channels. Vertical micro-pillars separated by 100µm populate the boundaries of each gel channel with the corresponding media. This configuration allows surface tension effects during the filling of cell-laden hydrogels and paracrine interactions between endothelial cells and flanking HLF [41]. In such a culture system, endothelial cells self-assemble into µVN through a vasculogenesis process. Thus, endothelial cells form vacuoles and establish connections as early as few hours after the seeding. Further maturation of microvascular structures with tubulogenesis and lumen formation usually requires more than 48 hours of cultures. These structures are stable up to one week [41]. For visualization of these structures, cells were fixed with 4% PFA and stained using standard immunofluorescent protocols [41]. Acquisition and visualization are done by confocal microscopy. The analyses were performed on 3D microvascular networks after 24 hours of *in vitro* culture, when network connections are fully established. Quantifications were obtained using a freely available imaging analysis tool angiogenesis analyzer, freely available ImageJ software, version:2.1.0/1.53c, http://imagej.net/contributors applied on 2D maximum projected confocal stacks of fluorescent signal from phalloidin staining. These values were normalized taking into account the image size.

Wound healing assay

Cell migration was evaluated using a scratch wound assay. Twenty thousand sub-confluent EC-CTEPH and HPAE were seeded in 24-well plates and starved prior to scratching the cell monolayer with a p200 pipette tip to generate a wound. Non-adherent cells were removed by washing and normal growth medium was added for 48h. Pictures were taken at baseline and 8h, 24h, 32h and 48h. Wound closure was expressed as percentage of regrowth divided by area and width of original wound. The healing area was analyzed with freely available imaging processing ImageJ software, version:2.1.0/1.53c, http://imagej.net/contributors.

Subcutaneous Sponge Implantation Assay for *in vivo* Vascularization

Male non-obese diabetic (NOD) severe immunodeficiency genetic disorder (SCID)-IL-2 gammaRnull mice aged 10–12 weeks were bred and maintained in the animal facilities of the University of Barcelona. All procedures were conducted following the European Directive 2010/63/UE and Spanish RD 53/2013 regulations related to the Guide for the Care and Use of Laboratory Animals and in compliance with the ARRIVE guidelines. The study protocol was approved by the Animal Experimentation Ethics Committee of the University of Barcelona (DAAM 10028).

Anesthetic comprised Ketamina (100mg/ml) and Medetomidina (1mg/ml), given intraperitoneally at a single dose of 7.5ul/10 gbw and 10ul/10 gbw. Reversal of anesthesia was induced, after at least 20 minutes of unconsciousness, using Atipamezole (5mg/ml) in water for injection. This was given subcutaneously at a single dose of 2ul/10 gbw. Meloxicam was given subcutaneously after surgery (2mg/ml) at 10ul/10 gbw. Mice were anesthetised and a sterilised sponge cylinder (0.5 cm^3) (Caligen Foam) was implanted subcutaneously on each flank. Each animal had a control vehicle-impregnated sponge implanted on one flank and cell-impregnated sponge on the other flank. Each animal had a control vehicle-impregnated sponge (growth-factor-reduced [GFR]-Matrigel alone) implanted on one flank and cell-impregnated sponge (GFR-Matrigel plus CTEPH-EC or

HPAE) on the other flank. Sponges were impregnated with 1x10^5 cells cells/mL of CTEPH-EC or HPAE in complete EGM-2 medium and mixed with 250µL of GFR-M. Mice were humanely euthanized 21 days following implantation with overdose of anesthesia by intraperitoneal single dose (100 mg/Kg. Stock solution 200 mg/ml) of sodium Pentobarbital. Confirmation of death was carried out by cervical dislocation. Sponges were fixed in 4% PFA before embedding in paraffin wax. Sections (5µm) were stained with H/E for identification of blood vessels, as described [42]. Vessel density within sponges was determined using the mean of triplicate vessel counts on each of two sections per sponge.

Electron microscopy

CTEPH-EC or HPAE were washed twice with PBS and fixed with 2.5% (w/v) glutaraldehyde in 0.1 M cacodylate buffer (Electron Microscopy Sciences) for 10 min at RT. Cells were recovered by scraping and centrifuged at 1200rpm 4°C for 4 min. Cell pellets were stored at 4°C and analyzed by the scientific and technologic center of University of Barcelona.

High resolution respirometry (OROBOROS)

Oxygraph-2k (Oroboros Instruments) was used to study cellular respiratory metabolism. This system is composed of two chambers for cell loading and two electropoles for sensing the consumption of oxygen in each chamber. DataLab software was used to calculate results based on the number of cells introduced and on the protein concentration. Calibration prior to each experiment was required following the manufacturer's instructions. 1x10^6 CTEPH-EC or HPAE per ml were resuspended in 100µl of MiR05 medium and introduced into one of the chambers at a final volume of 2ml. Two different assays were run in parallel testing the respiratory flux control of both HPAE and CTEPH-EC simultaneously. i) Respiratory capacity assessment: First initial monitoring of endogenous cell respiration (routine) was measured. Cells were then subjected to different exogenous compounds -0.6µl of oligomycin (0.25mM) (inhibitor of complex V) as an indicator of proton leakage, increasing concentrations of CCCP (1mM) until respiration no longer increased, indicative of maximal respiratory capacity, 0.25µl of antimycin (0,2mM) (inhibitor of complex III) was added to end the assay by completely inhibiting respiration. ii) Complex I, II, III and IV were also analyzed using specific substrates and inhibitors allowing the different complexes to be analyzed separately (see Supplementary Table 4 for details). All data was recorded using DataLab software v5.1.1.9 (Oroboros Instruments). Results were expressed as median and as 25% and 75% percentile, statistical analysis was performed with GraphPad Prism 7 software, version 7.0e, serial number:GP7-0633739-R###-#####, https://www.graphpad.com.

Mitochondrial morphology and content

Immunocytochemistry was performed as previously described using confocal microscopy [43]. One cell from three different fields for each cell line was randomly selected and analyzed with Image J software to quantify the following parameters of mitochondrial dynamics: i) Mitochondrial content: Total number of mitochondria/total cell area; ii) Circularity (Circ): 4π.area/perimeter^2; circular mitochondria have fewer interaction sites with other mitochondria, thus, Circ=1 refers to poor mitochondrial dynamics of isolated mitochondria [43]. iii) Aspect ratio (AR) or mitochondrial elongation: major/minor axis, AR = 1 indicates a perfect circle; AR increases as mitochondria elongate and become more elliptical, considered a beneficial sign of mitochondrial dynamics.

Mitochondrial content was also determined using mitotracker green (MTG) following manufacturer´s instructions. Briefly, a total of 1 ml of complete culture media containing roughly $2x10^5$ cells was prepared for different reaction procedures and incubated: (i) in the absence of any dye as control for autofluorescence, (ii) with 200nM MTG fluorophore (Molecular Probes) for 30 min. Cytometric analyses were performed using a FACScalibur cytometer (Becton Dickinson). Results were expressed as median or percentage of cells with specific fluorescence.

Detection of oxidative stress

Cellular oxidation in HPAE and CTEPH-EC was measured using cell-permeant CellROX Deep Green reagent (ThermoFisher Scientific) following manufacturer's instructions. 250ul/well of 5 µM CellROX was added to cells seeded in triplicate at 80% confluence in μ-Slide 8 Well (Ibidi) and incubated for 30 min at 37°C. Cells were washed three times with HBSS/Ca/Mg buffer and fixed with 3.7% formaldehyde for 15 min before analysis using fluorescence microscopy 485/520nm. Nuclei were stained with blue-fluorescent Hoechst 33342. MitoSOX, mitochondrial Superoxide Indicator (ThermoFisher Scientific) was used to detect generation of the mitochondrial superoxide anion following manufacturer's instructions. 5mM MitoSOX reagent stock solution was diluted in HBSS/Ca/Mg to make a 5µM MitoSOX working solution. 250ul/well of 5 µM MitoSOX was added in triplicate in a 80% confluent μ-Slide 8 Well (Ibidi). Cells were incubated for 10 min at 37°C, washed three times with HBSS/Ca/Mg buffer and analyzed using fluorescence microscopy 640/665 nm. Nuclei were stained with blue-fluorescent Hoechst 33342.

Oxyblot

Total oxidized protein content was measured with the Oxyblot Protein Oxidation Kit (Merck Millipore) following manufacturer's instructions**.** Briefly, 20µg of protein samples were mixed with an equal volume of 12% SDS and then incubated with an equal volume of 1X dinitrophenylhydrazine (DNPH) derivation solution at RT for 15 min before addition of neutralization solution to terminate the reaction. The DNPH-tagged proteins were then used for SDS-PAGE/Western blot and loaded directly onto a PVDF membrane. An anti-DNP antibody was used for detection of the DNPH-tagged proteins. The blots were developed using the SuperSignal West Dura Kit (ThermoFisher). The intensity of bands was quantified using freely available Image Lab software (Biorad laboratories), version 6.1.0 build7, http://www.biorad.comand analyzed by freely available imaging processing ImageJ software, version:2.1.0/1.53c, http://imagej.net/contributors.

Permeability assay

To assess the extent of permeability in CTEPH-EC compared to HPAE cells, endothelial cells were seeded into collagen-coated inserts (Millipore *In Vitro* Vascular Permeability Assay) until a confluent monolayer is formed. After that, following the manufacturer´s instructions, a high molecular weight FITC-Dextran is added on top of the cells, to allow the fluorescent molecules to pass through the endothelial monolayer at a rate proportional to the monolayer´s permeability. The levels of permeability were quantified using multiwell spectrophotometer to measure absorbance of the dye solution at 535 nm.

Statistical analysis

Statistical analyses were performed using GraphPad Prism 7 software, version 7.0e, serial number:GP7-0633739-R###-#####, https://www.graphpad.com. Data are shown as

mean ± SD. Pairwise comparisons between patients and controls were performed using Mann Whitney U test for non-normally distributed variables. Comparisons involving more than two groups were performed with Kruskal-Wallis One Way Analysis of Variance and when significant, post-hoc Dunn's Multiple Comparison Test. A two-way ANOVA followed by Tukey´s multiple comparisons test was used to analyze cellular perimeter, area, and diameter for the two sample groups at different time points. Spearman rank correlation coefficient was used as a hypothesis test to study the dependence between two random variables. Statistical significance was assumed if $p \leq 0.05$ (for a confidence interval of a=95%).

SUPPLEMENTARY REFERENCES

15. Lau EM, Tamura Y, McGoon MD, Sitbon O. The 2015 ESC/ERS Guidelines for the diagnosis and treatment of pulmonary hypertension: a practical chronicle of progress. *Eur Respir J* 2015;**46**:879-882.
16. Duim SN, Kurakula K, Goumans MJ, Kruithof BP. Cardiac endothelial cells express Wilms' tumor-1: Wt1 expression in the developing, adult and infarcted heart. *J Mol Cell Cardiol* 2015;**81**:127-135.
39. Tura O, Barclay GR, Roddie H, Davies J, Turner ML. Absence of a relationship between immunophenotypic and colony enumeration analysis of endothelial progenitor cells in clinical haematopoietic cell sources. *J Transl Med* 2007;**5**:37.
17. Ingram DA, Mead LE, Tanaka H, Meade V, Fenoglio A, Mortell K, Pollok K, Ferkowicz MJ, Gilley D, Yoder MC. Identification of a novel hierarchy of endothelial progenitor cells using human peripheral and umbilical cord blood. *Blood* 2004;**104**:2752-2760.
40. Chiu CH, Lei KF, Yeh WL, Chen P, Chan YS, Hsu KY, Chen AC. Comparison between xCELLigence biosensor technology and conventional cell culture system for real-time monitoring human tenocytes proliferation and drugs cytotoxicity screening. *Journal of orthopaedic surgery and research* 2017;**12**:149.
41. Chen MB, Whisler JA, Frose J, Yu C, Shin Y, Kamm RD. On-chip human microvasculature assay for visualization and quantification of tumor cell extravasation dynamics. *Nat Protoc* 2017;**12**:865-880.
42. Tura O, Skinner EM, Barclay GR, Samuel K, Gallagher RC, Brittan M, Hadoke PW, Newby DE, Turner ML, Mills NL. Late outgrowth endothelial cells resemble mature endothelial cells and are not derived from bone marrow. *Stem Cells* 2013;**31**:338-348.
43. Juarez-Flores DL, Gonzalez-Casacuberta I, Ezquerra M, Bano M, Carmona-Pontaque F, Catalan-Garcia M, Guitart-Mampel M, Rivero JJ, Tobias E, Milisenda JC, Tolosa E, Marti MJ, Fernandez-Santiago R, Cardellach F, Moren C, Garrabou G. Exhaustion of mitochondrial and autophagic reserve may contribute to the development of LRRK2 (G2019S) -Parkinson's disease. *J Transl Med* 2018;**16**:160.

Characteristics	Data
Age, years	62.5±6.5
Male sex, n (%)	8 (53.3%)
BMI, kg/m^2	27.2±3.7
mPAP, mmHg	38.3±6.8
PVR, $din \cdot s \cdot cm^{-5}$	576.1±217.5
PAOP, mmHg	9.3±4.3
BNP, pg/ml	137.2 ± 280.7
CI, $L/min/m^2$	2.2 ± 0.4
RAP, mmHg	6.7 ± 4.3
SvO_2, %	6.2 ± 6.0
WHO FC, n (%)	
I	1 (7.1%)
II	3 (21.4%)
III	10 (71.4%)
6MWT, m	434.0 ± 81.7
Survival, yrs	3.0 ± 3.0

Supplementary Table 1. Clinical characteristics, lung function and laboratory measurements

Definition of abbreviations: Body mass index (BMI), Mean pulmonary arterial pressure (mPAP), pulmonary vascular resistance (PVR), The pulmonary artery occluded pressure (PAOP), Brain natriuretic peptide (BNP), Cardiac Index (CI), Right Atrial Pressure (RAP), Pulmonary arterial oxygen saturation ($SV0_2$), World Health Organization

functional class (WHO-FC) and 6-minute walk distance (6MWD). CTEPH (n=14), values expressed as mean ± SD.

Table Antibodies

Flow cytometry AB			
**Name**	**Fluorochrome**	**Company**	**cat number**
CD144	PE	BD Pharmingen	560410
CD34	PECy7	eBioscience	25-0349-42
CD146	FITC	BD Pharmingen	560846
CD62	APC	BD Pharmingen	551144
KDR, VEGFR2	PerCP/Cy5.5	affymetrix (eBioscience)	45-0459
CD45	FITC	BD Pharmingen	345808
CD133	PE	BD Pharmingen	555473
CD42b	alexa fluor	Biolegend	135117
C-Kit, CD117	brilliant Violet 421TM	Biolegend	313215
CD56	FITC	Abcam	ab8211
SMA	FITC	BD Pharmingen	555445
CD31	FITC	Biolegend	323203
CD105	FITC	Sigma	L9006
UEA-1	FITC	BD Pharmingen	561712
**Immunofluorescence**			
**Name**	**Host**	**Company**	**cat number**
a-SMA	mouse	Dako	M0851
vwf	rabbit	abcam	ab6994
CD31	mouse	Dako	M0823
8-hydroxiguanosine (8-OH-dG)	rabbit	Bioss antibodies	BS-1278R
UEA-1 Ulex	Ulex europaeus	Sigma	L9006
vwf	rabbit	abcam	ab6994
ve-cad (CD144)	mouse	BD Pharmingen	555661
eNOS	mouse	abcam	ab76198
Calponin	mouse	Dako	M3556
Ki-67	mouse	Leika	ack02
**Western Blot**			
**Name**	**Host**	**Company**	**cat number**
CD31	mouse	Dako	M0823
VWF	rabbit	abcam	ab6994
CAV1	mouse	santa cruz biotechnology	sc-53564
eNOS	mouse	abcam	ab76198
MFN1	mouse	santa cruz biotechnology	sc-166644
MFN2	mouse	abcam	ab56889
OPA1	mouse	bd bioscience	bd612606
DRP1	mouse	santa cruz biotechnology	sc271583
SOD1	rabbit	SAB Signalway Antibody	32058
SOD2	rabbit	SAB Signalway Antibody	32265
Jagged1	rabbit	abcam	ab7771
NOTCH1	mouse	santa cruz biotechnology	sc-373891
Dll4	rabbit	abcam	ab176876

Supplementary Table 2: Antibodies used in this study

Primer Name	Forward primer (5'-3')	Reverse primer (5'-3')
**VE-CAD**	GATGCAGACGACCCCACTGT	CCACGATCTCATACCTGGCC
**CD31**	AAAGTCGGACAGTGGGACGT	GGCTGGGAGAGCATTTCACA
**ANG1**	AATATGCCAGAACCCAAAAAG	CAATATTCACCGGAGGGATTT
**Myocardin**	ACAGCGCGGTTTTTTCCA	CACCGAGGAACACGGAGC
**vWF**	CCTTGAATCCCAGTGACCCTGA	GGTTCCGAGATGTCCTCCACAT
**eNOS**	GGCCCGGATCCAGTGGG	GTGGTTGCAGATGTAGGTGAACA
**CAV1**	CATCCCGATGGCACTCATCTG	TGCACTGAATCTCAATCAGGAAG
**VEGF**	GCCTTGCTGCTCTACCTCCAC	ATGATTCTGCCCTCCTCCTTCT
**ANG2**	TTCCTCCTGCCAGAGATGGA	TGCACAGCATTGGACACGTA
**CD44**	TCCAACACCTCCCAGTATGACA	GGCAGGTCTGTGACTGATGTACA
**ICAM-1**	CAGAGGTTGAACCCCACAGT	CCTCTGGCTTCGTCAGAATC
**VCAM-1**	GCAAAGGGAGCACTGGGTTGACT	GCCACATTGGGAAAGTTGCACAGG
**HOXD3**	CGTAAGGATTGCATCGGACT	TCCTAAGCTCGGCTGGATAA
**HOXD8**	TAAACCAGCTTGCTGTGTGC	GTGAGGCTATCGCTTTCCTG
**HOXD9**	CCTGCTCCATTGGTTCCTTA	TCAGAAACATGGGGGACATT
**Cas3**	AGGACTCAAATTCTGTTGCCACC	TGGAACAAATGGACCTGTTGACC
**Cas8**	GATTTGCTGATTACCTACCTAAACACT	TCTGAAATCTGATAGAGCATGACC
**Cas9**	ACACCCAGTGACATCTTTGTGT	GTCTCAACGTACCAGGAGCC
**p21**	CTGGAGACTCTCAGGGTCGAA	GGCGGATTAGGGCTTCCTC
**p53**	GAGCTGAATGAGGCCTTGGA	CTGAGTCAGGCCCTTCTGTCTT
**BCL2**	GGGAGGATTGTGGCCTTCTT	CAGGTACTCAGTCATCCACA
**MFN1**	TCTGGGCCTGATGAGGGTAA	TTCCTCCCAGGAGCTCCTAC
**MFN2**	CACAAGGTGAGTGAGCGTCT	TCCATGTACTCGGGCTCTGA
**OPA1**	TGCCTGACATTGTGTGGGAAA	TTCCGGAGAACCTGAGGTAA
**DRP1**	CACCCGGAGACCTTCTCATTC	CCCCATTCTTCTGCTTCCAC
**SOD1**	GGTGGGCCAAAGGATGAAGAG	CCACAAGCCAAACGACTTCC
**SOD2**	GCCCTGGAACCTCACATCAA	TCAGGTTGTTCACGTAGGCC

Supplementary Table 3: Primer sequences used in this study

Complex	Substance	Inhibitor/Substrate
I	20ìl of glutamate (0.5M)	substrate
I	8ìl of malate (0.5M)	substrate
I	40ìl of ADP (500mM)	substrate
I	40ìl of pyruvate (250mM)	substrate
I	1ìl of rotenone (0.5mM)	inhibitor
II	20ìl of succinate (1M)	substrate
II	10ìl of malonate (10mM)	inhibitor
III	43ìl of glyceraldehyde 3-phosphate (G3P) (0.5M)	substrate
III	25ìl of antimycin (0.2mM)	inhibitor
III	43ìl of ascorbate (5mM)	substrate
IV	43ìl of N,N,N′,N′-tetramethyl-p-phenylenediamine (TMPD) (1mM)	substrate
IV	17ìl of potassium cyanide (80mM)	inhibitor

Supplementary Table 4: Complex I, II, III and IV specific substrates and inhibitors.

% of expression	Mean±SD
**Endothelial markers**	
CD34+	6.7±4.2
CD144+	4.9±2.3
CD31+	10.9±7.7
CD146+	7.6±8.1
KDR+	1.6±2.2
**Endothelial and Mesenchymal markers**	
CD105+	25.76±6.1
CD56+	2.5±3.2
Tie2+	24.0±5.2
**Muscular marker**	
α-SMA+	11.8±5.2
**Progenitor markers**	
CD133+	1.2±1
**Leukocyte marker**	
CD45+	11.1±8.2
CD14+	0,05±0,21
CD16+	0,04±0,15
**Platelet marker**	
CD62+	1.2±1.3
CD42b+	1.8±2.1
**Morphometric measurements**	
Neointima / % of total thickness	89.17±3.94
Neointima / % of total area	87.17±3.21
Number of microvessels (mm^2/tissue)	47.5±14.2

Supplementary Table 5: A collagen digested PEA material was analyzed by flow cytometry. Percentage of expression is given as mean ± SD. PEA material presented an enlarged neointima and the presence of microvascular vessels.

Supplementary Figures and legends

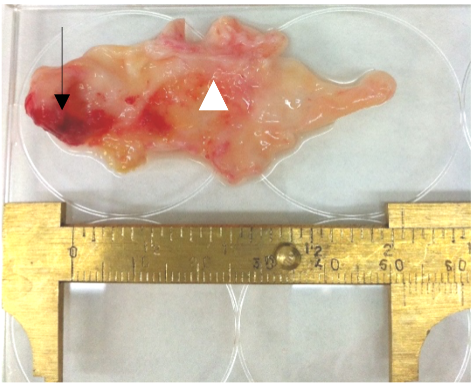

Supplementary Fig. 1: Specimens collected during pulmonary endarterectomy (PEA) from a patient with CTEPH. The presence of a thrombus is indicated by the black arrow and the tissue used for isolation of pulmonary artery ECs (cell culture) is indicated by the white arrowhead.

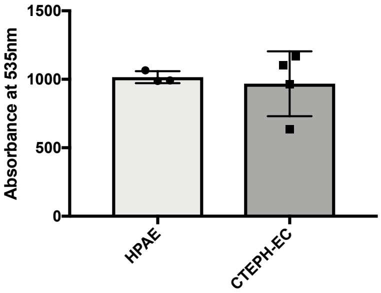

Supplementary Fig. 2. Permeability assay. Permeability levels between CTEPH-EC and HPAE. Values expressed as mean ± SD, n=3 HPAE, n=4 CTEPH independent experiments, $p>0.05$, Mann Whitney *U* test. Statistical analysis was performed with GraphPad Prism 7 software, version 7.0e, serial number:GP7-0633739-R###-#####, https://www.graphpad.com.

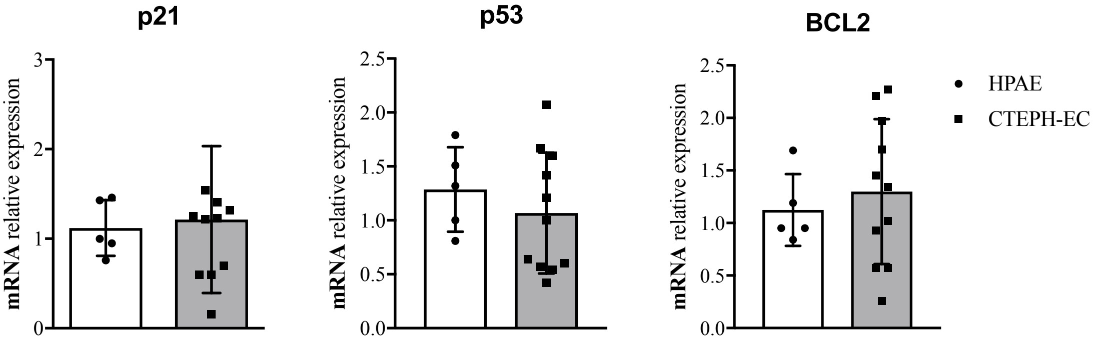

Supplementary Fig. 3: **Apoptosis in CTEPH-EC.** mRNA expression profile of apoptotic related markers p21, p53 and BCL2 in CTEPH-EC and HPAE. CTEPH-EC, n=11; HPAE, n=5 independent experiments performed in triplicate $p>0.05$, Mann–Whitney *U* test, values expressed as mean ± SD. Statistical analysis was performed with

GraphPad Prism 7 software, version 7.0e, serial number:GP7-0633739-R###-#####, https://www.graphpad.com.

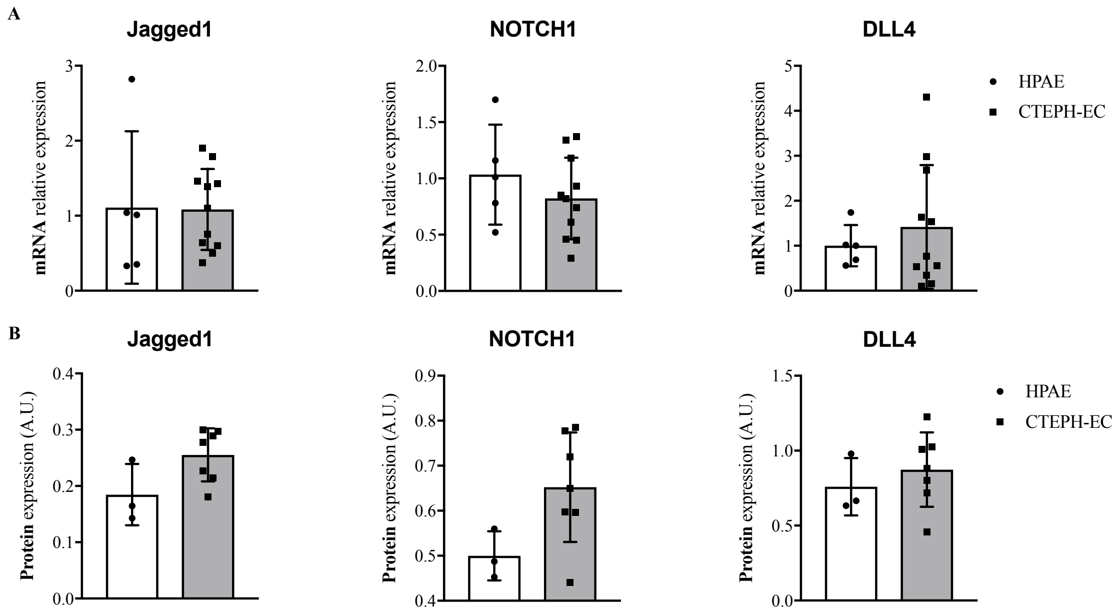

Supplementary Fig. 4. Notch signaling pathway in CTEPH-EC. A-B) mRNA and protein expression of Notch1 and its ligands DLL4 and Jagged1 in CTEPH-EC and HPAE. CTEPH-EC. CTEPH-EC, n=11; HPAE, n=5 independent experiments performed in triplicate for mRNA studies and CTEPH-EC, n=7; HPAE, n=3 independent experiments for protein studies, p>0.05, Mann–Whitney *U* test, values expressed as mean ± SD. Statistical analysis was performed with GraphPad Prism 7 software, version 7.0e, serial number:GP7-0633739-R###-#####, https://www.graphpad.com.

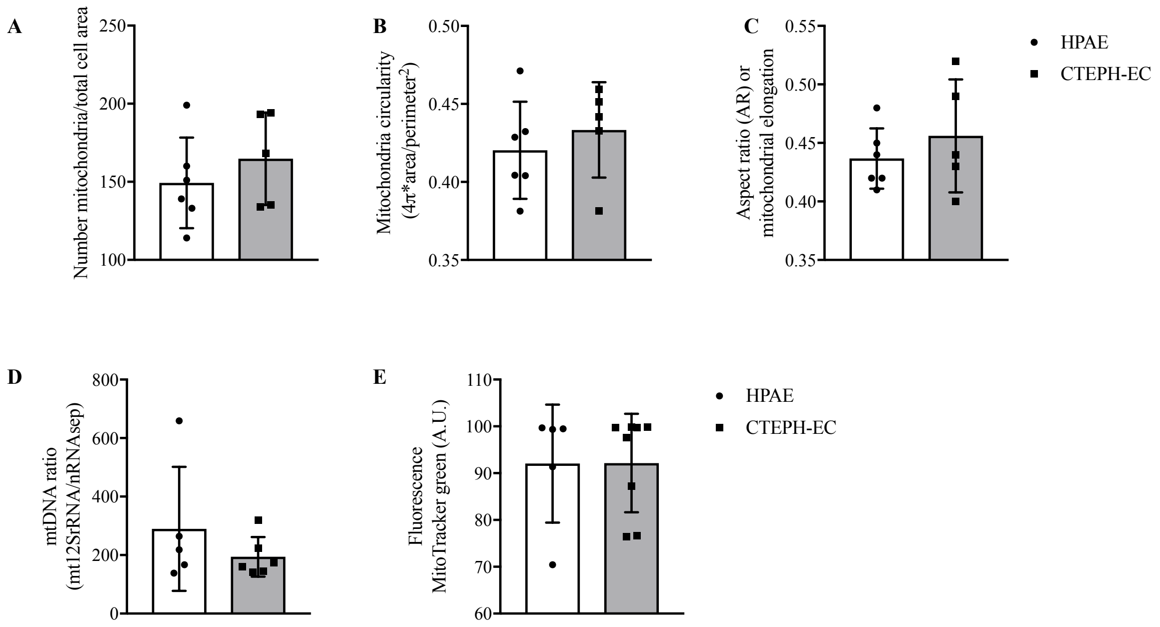

Supplementary Fig. 5. A-C) Mitochondria in CTEPH-EC. The number of mitochondria over total cell area, mitochondrial circularity, and mitochondrial elongation in CTEPH-EC and HPAE. CTEPH-EC, n=5; HPAE, n=6 independent experiments, p>0.05, Mann–Whitney *U* test, values expressed as mean ± SD. **D)** mt12SrRNA gene/nRNAseP nuclear gene ratio in CTEPH-EC and HPAE. CTEPH-EC, n=6; HPAE, n=5 independent experiments performed in triplicate p>0.05, Mann–Whitney *U* test, values expressed as mean ± SD **E)** Mitochondrial DNA content measurement by the use of MitoTracker green in CTEPH-EC and HPAE, CTEPH-EC, n=8; HPAE, n=5 independent experiments, Mann–Whitney *U* test, values expressed as mean ± SD. Statistical analysis was performed with GraphPad Prism 7 software, version 7.0e, serial number:GP7-0633739-R###-#####, https://www.graphpad.com.

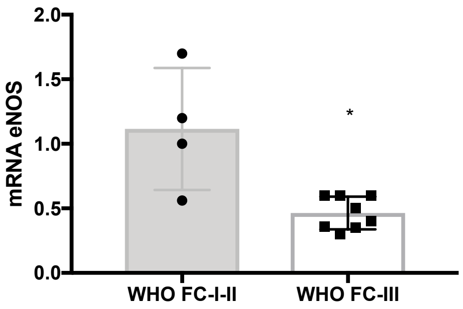

Supplementary Fig. 6. Correlation with clinical risk. Relationship between the levels of mRNA eNOS with World Health Organization functional class (WHO FC). CTEPH-EC, n=12; p=0.02*, Mann–Whitney *U* test, values expressed as mean ± SD. Statistical analysis was performed with GraphPad Prism 7 software, version 7.0e, serial number:GP7-0633739-R###-#####, https://www.graphpad.com**.**

Full-length gels represented in Figure 3B in order of appearance.

a)

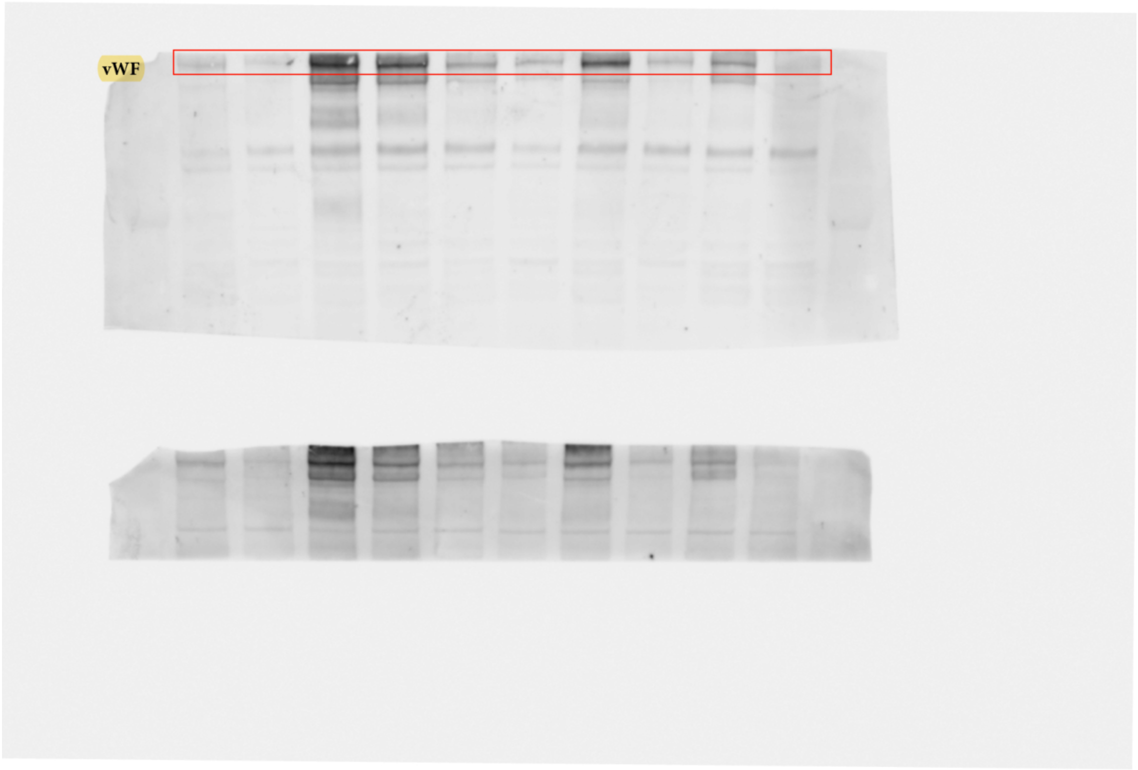

b)

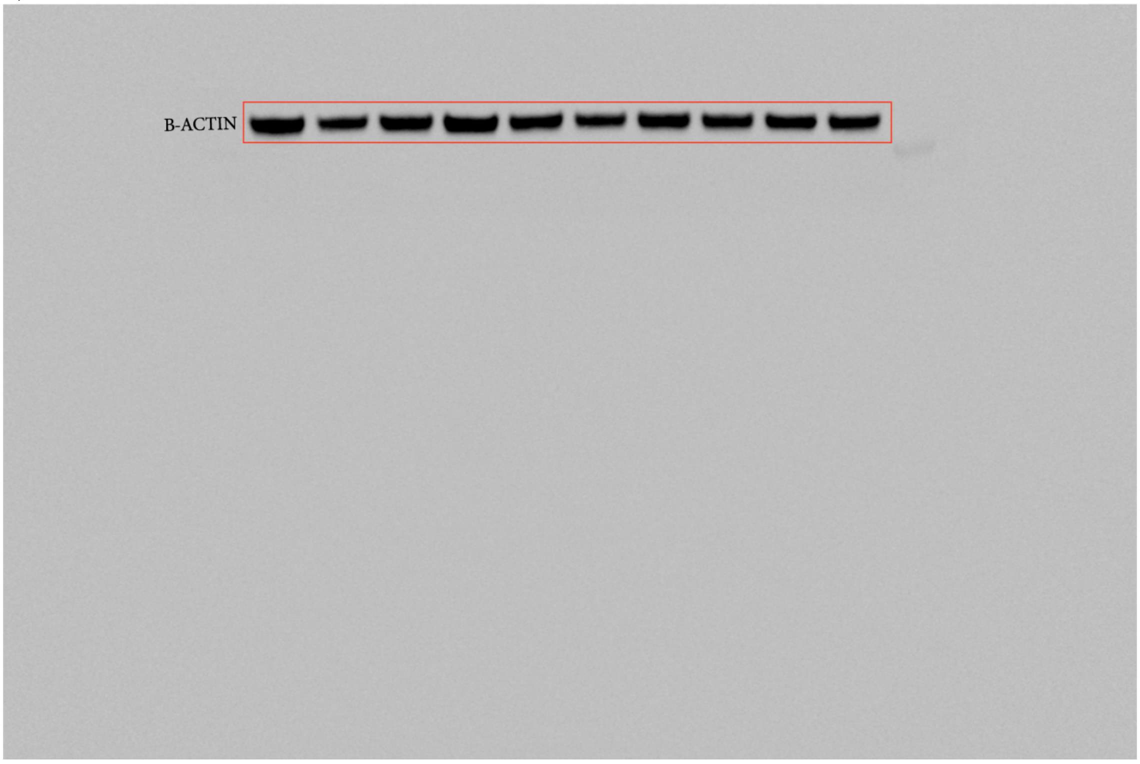

c)

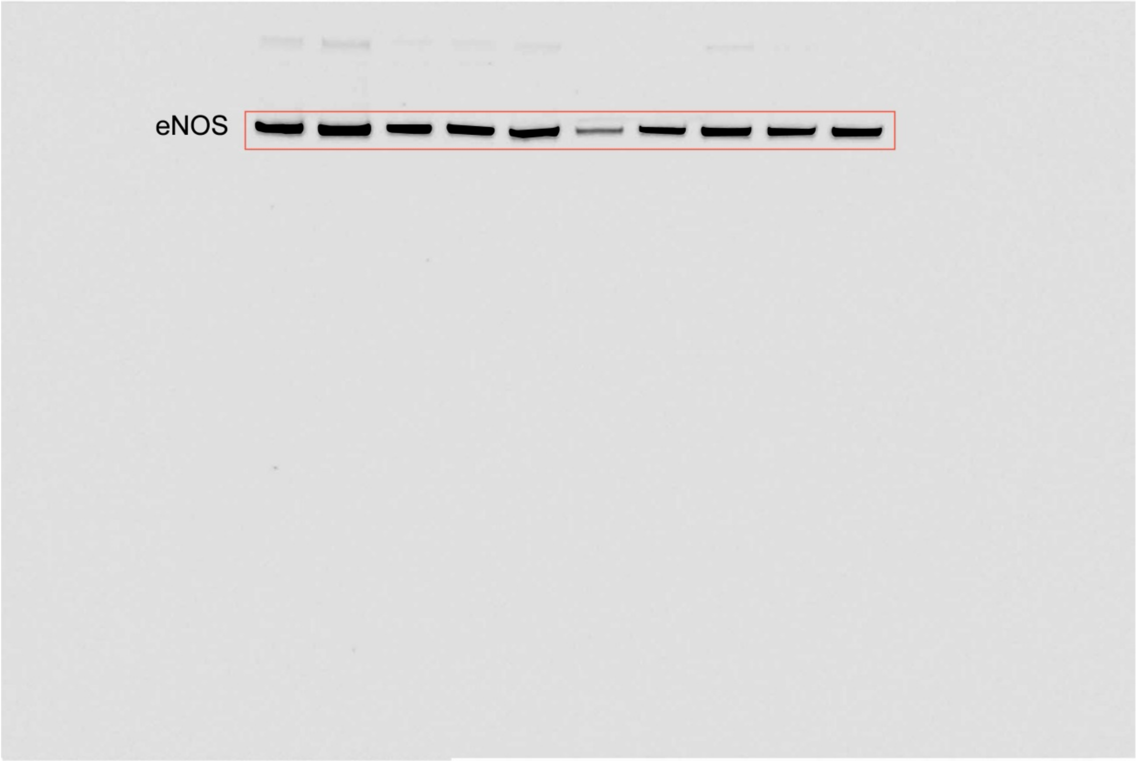

d)

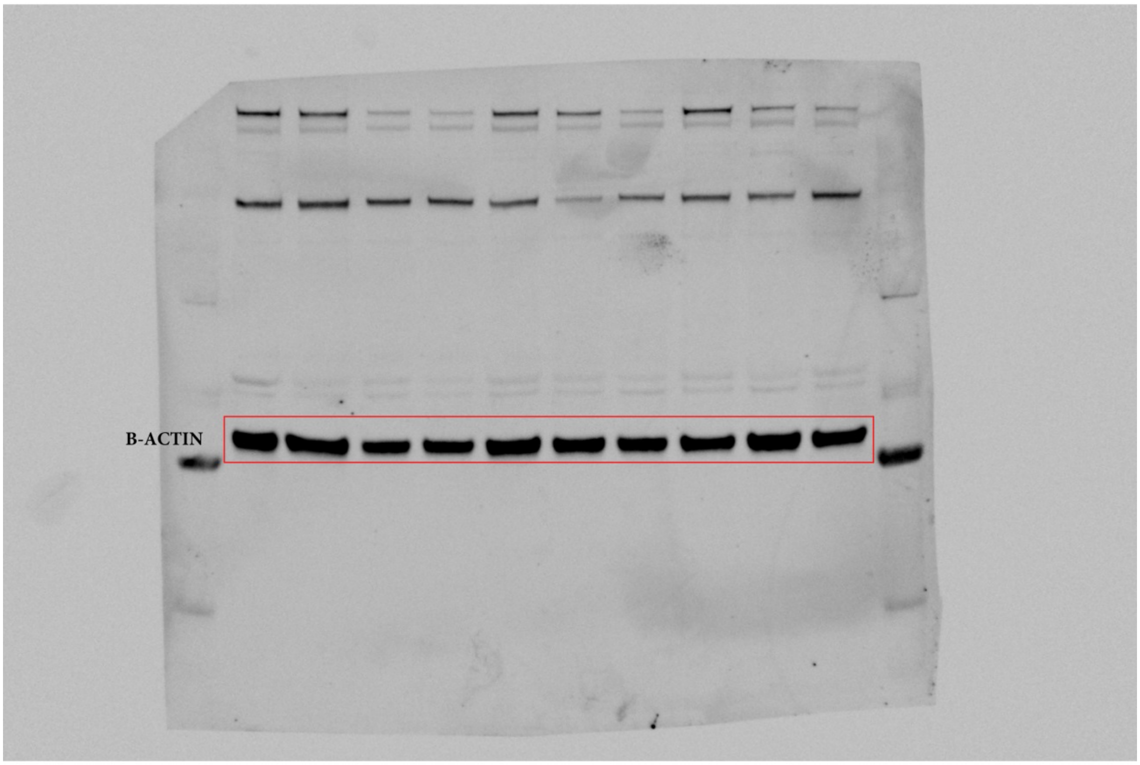

e)

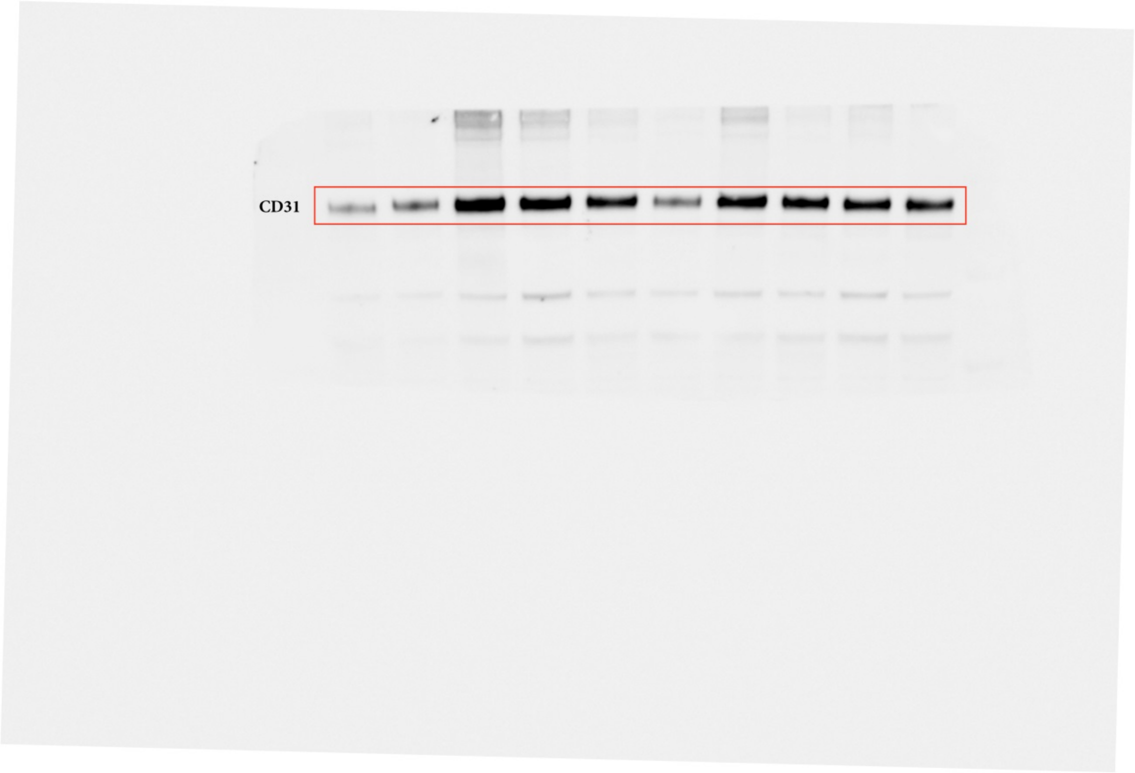

f)

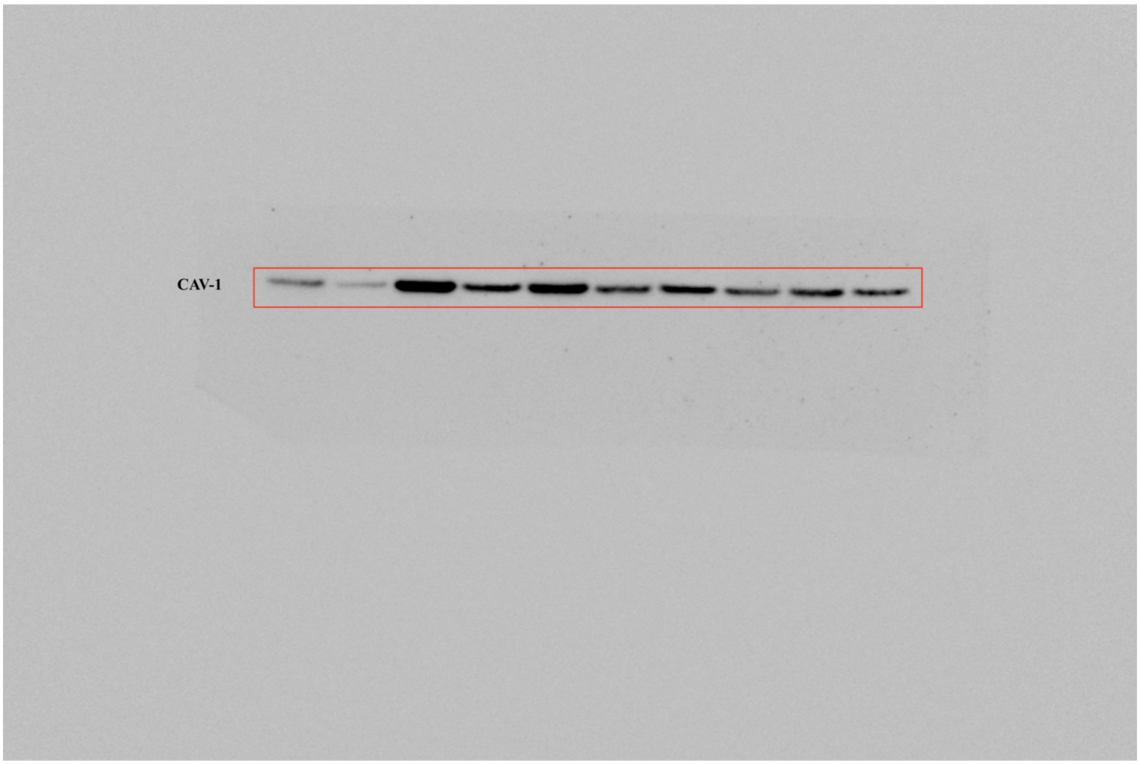

g)

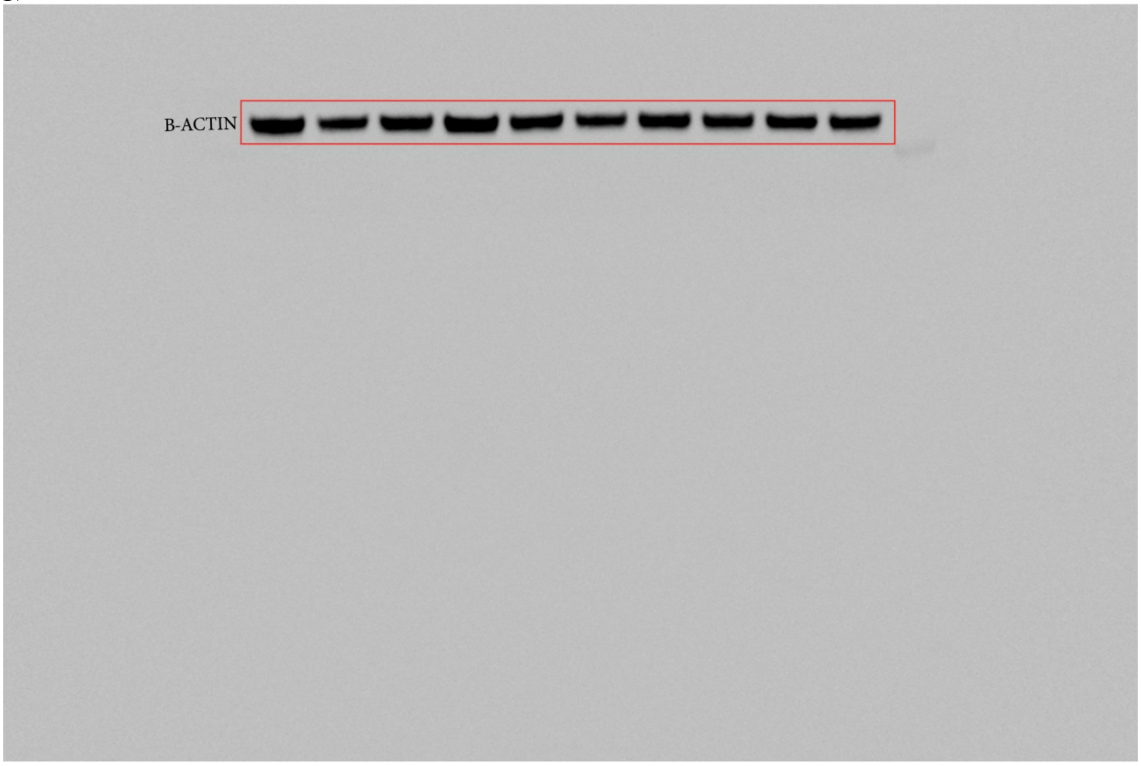

Supplementary Fig. 7. Full-length gels represented in main Figure 3B, a) vWF, b) ß-actin, c)eNOS, d) ß-actin, e)CD31, f) Cav-1 and g) ß-actin.

Full-length gels represented in Figure 7D in order of appearance.

a)

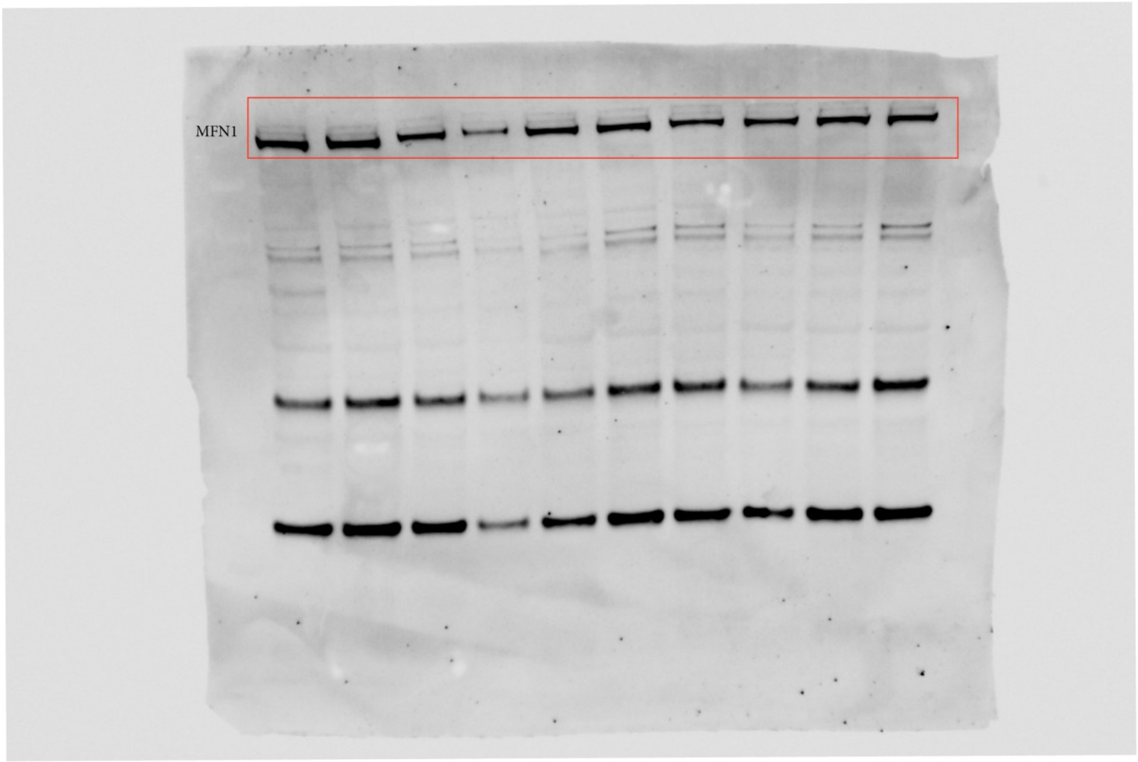

b)

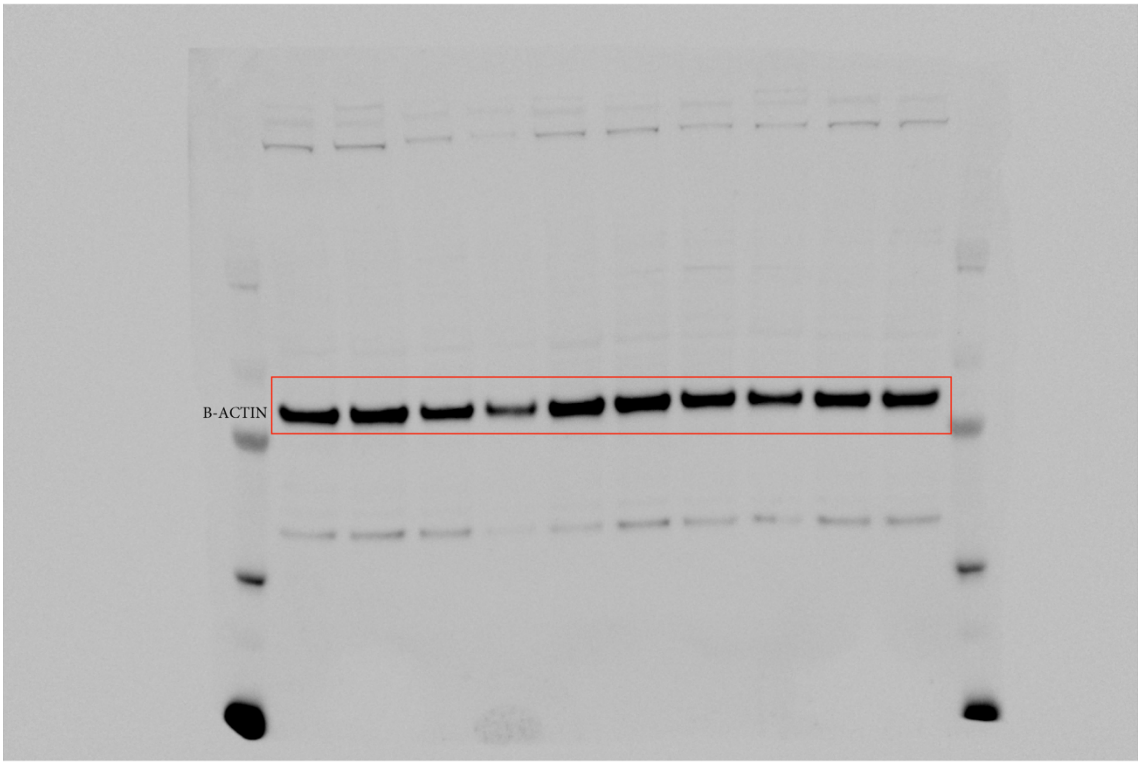

c)

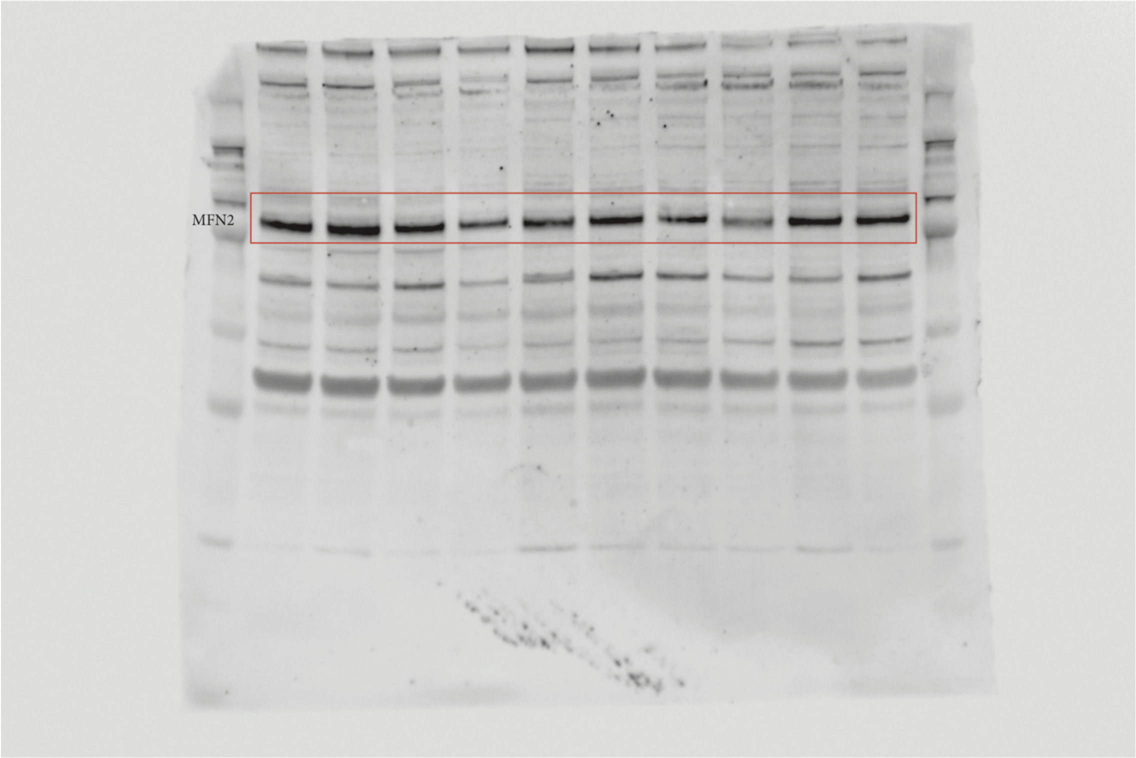

d)

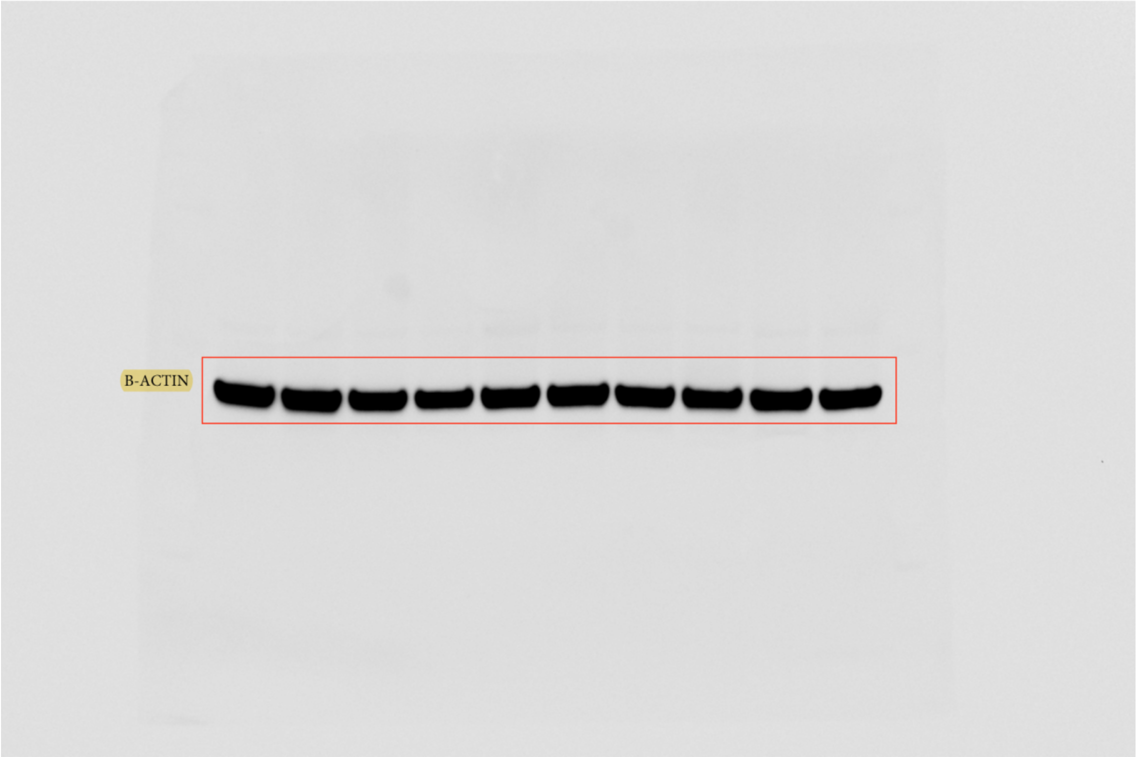

e)

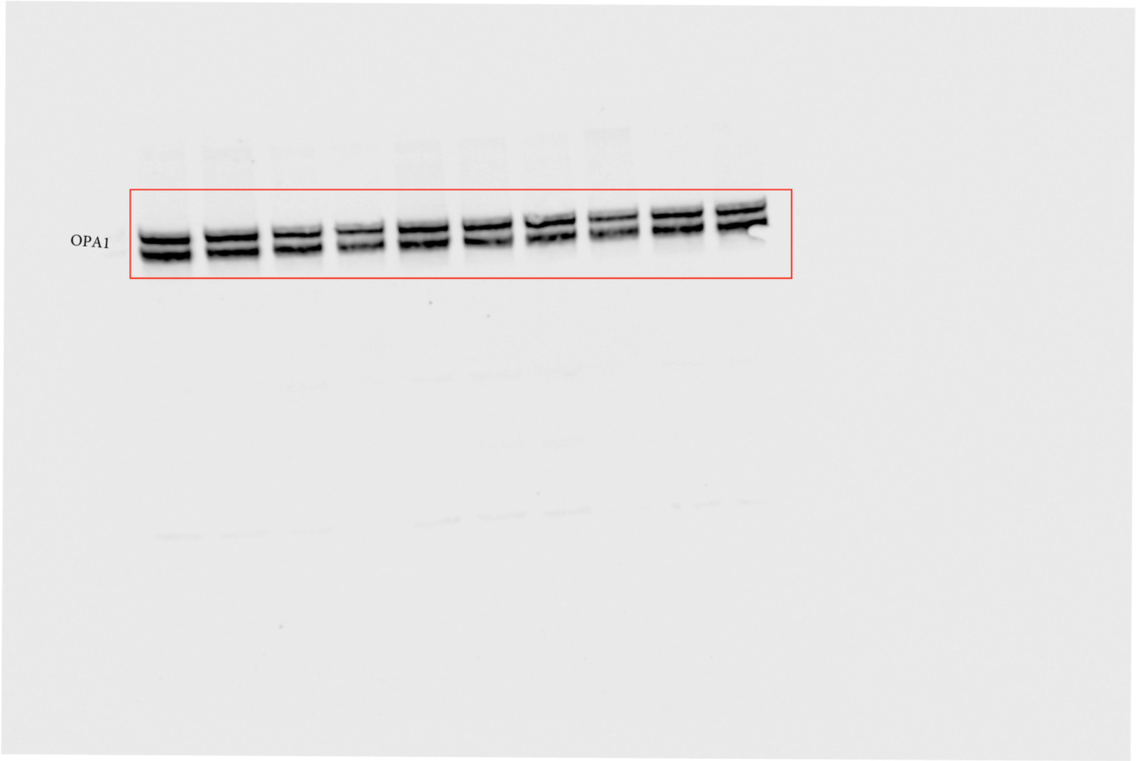

f)

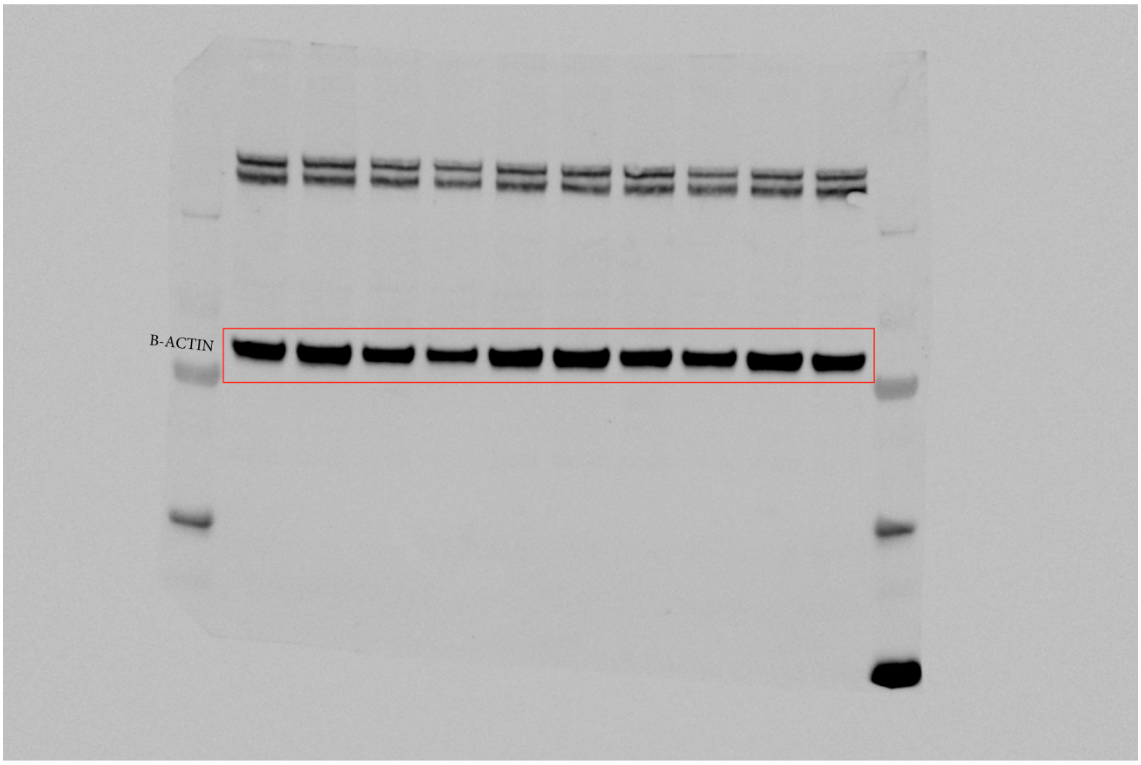

g)

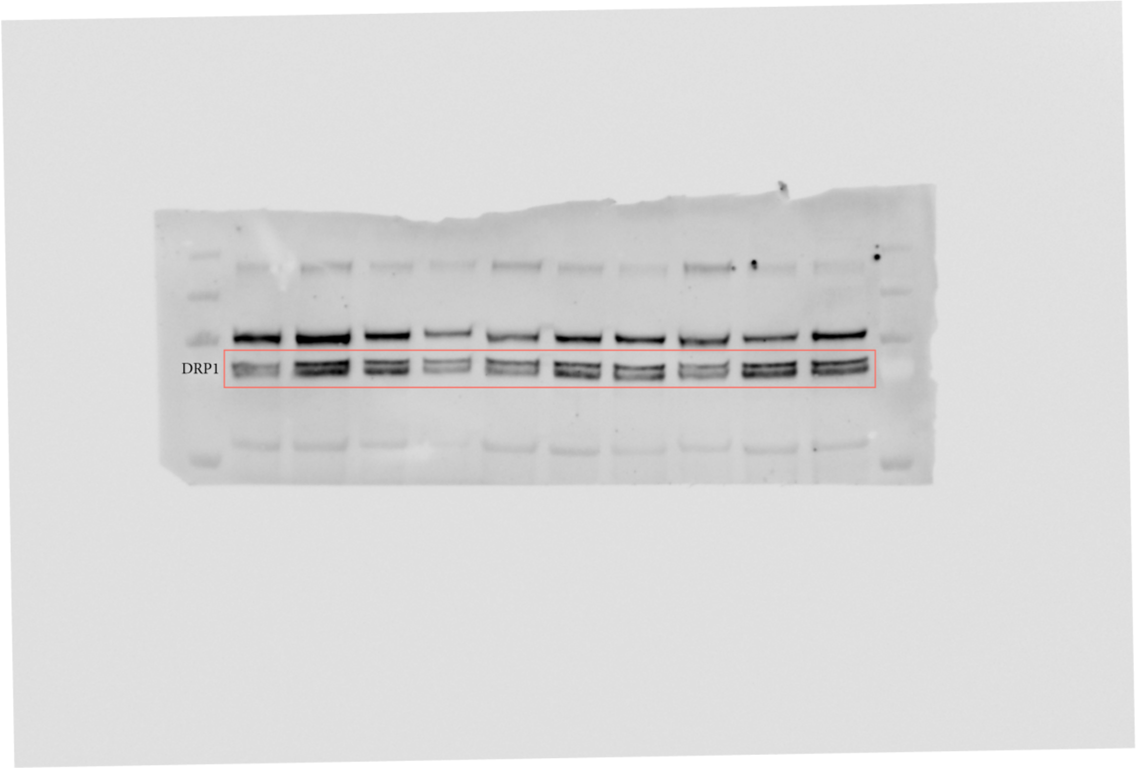

h)

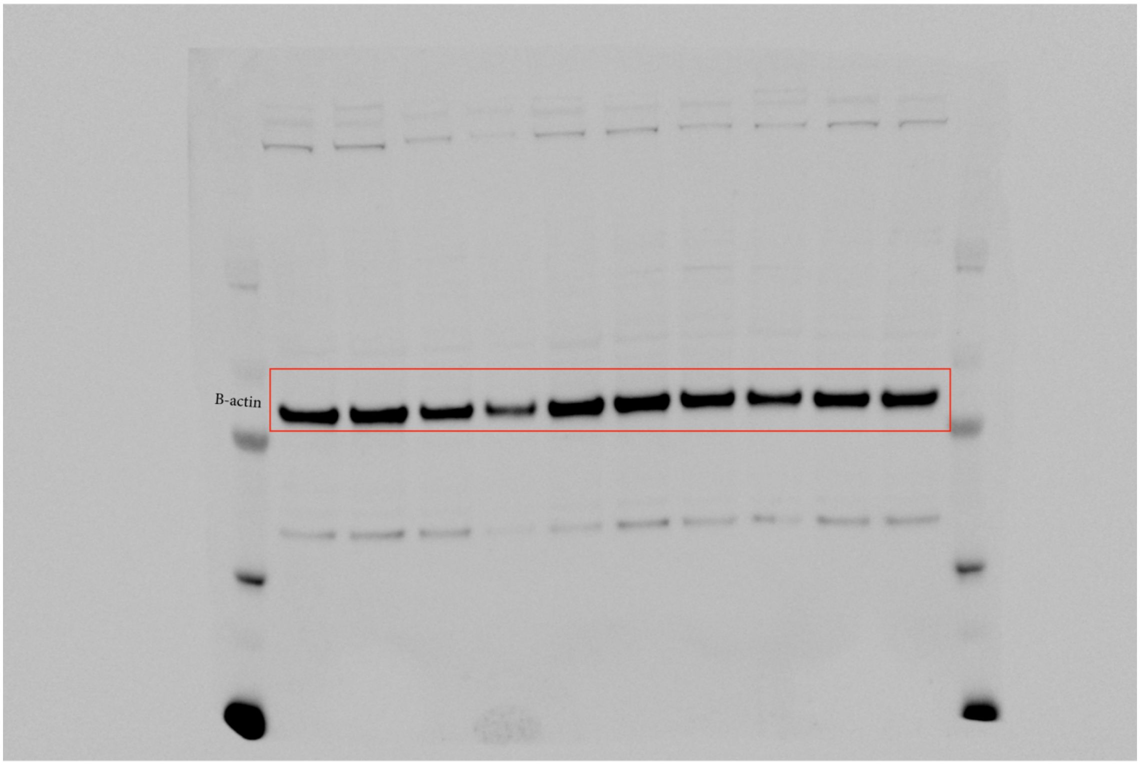

Supplementary Fig. 8. Full-length gels represented in main Figure 7D, a) MFN1, b) ß-actin, c)MFN2, d) ß-actin, e)OPA1, f) ß-actin, g) DRP1 and h) ß-actin.

Supplementary Video 1. 3D Projection of confocal stacks showing maturation of vasculogenesis of HPAE cells. The video is obtained from the confocal stacks of fixed samples 72 hours after seeding. Green=phalloidin; Blue=DAPI.

Supplementary Video 2. 3D Projection of confocal stacks showing maturation of vasculogenesis of CTEPH-EC. The video is obtained from the confocal stacks of fixed samples 72 hours after seeding. Green=phalloidin; Blue=DAPI.
